# Elevated O-GlcNAcylation induces an antidepressant-like phenotype and decreased inhibitory transmission in medial prefrontal cortex

**DOI:** 10.1038/s41598-020-63819-6

**Published:** 2020-04-24

**Authors:** Yoonjeong Cho, Hongik Hwang, Md. Ataur Rahman, ChiHye Chung, Hyewhon Rhim

**Affiliations:** 1 Center for Neuroscience, Brain Science Institute, Korea Institute of Science and Technology (KIST), Seoul, 02792 Republic of Korea; 2 Division of Bio-Medical Science and Technology, KIST School, Korea University of Science and Technology (UST), Seoul, 02792 Republic of Korea; 3 Department of Biological Science, Konkuk University, Seoul, 05029 Republic of Korea

**Keywords:** Depression, Prefrontal cortex, Molecular neuroscience, Synaptic transmission

## Abstract

Depression is a devastating mental disorder affected by multiple factors that can have genetic, environmental, or metabolic causes. Although previous studies have reported an association of dysregulated glucose metabolism with depression, its underlying mechanism remains elusive at the molecular level. A small percentage of glucose is converted into uridine diphosphate-*N*-acetylglucosamine (UDP-GlcNAc) via the hexosamine biosynthetic pathway, which serves as an immediate donor for protein O-GlcNAc modification. O-GlcNAcylation is a particularly common post-translational modification (PTM) in the brain, and the functional significance of O-GlcNAcylation in neurodegenerative diseases has been extensively reported. However, whether the degree of O-GlcNAc modification is associated with depressive disorder has not been examined. In this study, we show that increased O-GlcNAcylation levels reduce inhibitory synaptic transmission in the medial prefrontal cortex (mPFC), and that *Oga*^+/−^ mice with chronically elevated O-GlcNAcylation levels exhibit an antidepressant-like phenotype. Moreover, we found that virus-mediated expression of OGA in the mPFC restored both antidepressant-like behavior and inhibitory synaptic transmission. Therefore, our results suggest that O-GlcNAc modification in the mPFC plays a significant role in regulating antidepressant-like behavior, highlighting that the modulation of O-GlcNAcylation levels in the brain may serve as a novel therapeutic candidate for antidepressants.

## Introduction

Depressive disorder is a devastating mental disorder characterized by symptoms such as loss of interest, low mood, fatigue, and diminished cognitive functions^1–3^. A deficit in monoamine metabolism including serotonin, dopamine, and norepinephrine is known to contribute to its etiology^4–6^, and the regulation of synaptic function through monoamine neurotransmitters serves as the primary approach to treat patients with depressive disorder^7,8^. In addition, malfunctions of neuronal networks, such as an impaired balance between excitatory and inhibitory inputs, modulate emotional states, and previous studies suggest that glutamatergic and GABAergic systems are impaired in patients with depression^2,9,10^. Furthermore, altered GABAergic inhibitory function induced by a change in the expression levels of GABAergic receptors also disrupts emotional processes in animal models of depressive disorders^9–14^. Although the monoamine theory plays a major role in understanding the pathogenesis of depression, how the monoamine-independent changes in molecular and synaptic functions of neural networks contribute to depressive disorders remains largely elusive.

The medial prefrontal cortex (mPFC) underlies the executive control of animal behavior^15,16^, and structural and functional changes in the mPFC, such as synaptic transmission and altered protein expression levels, are implicated in depression-related behaviors in animal models^15–18^. For example, the deletion of the N-methyl-D-aspartic acid (NMDA) receptor subunit (GluN2B) in mPFC pyramidal neurons leads to antidepressant-like behavior in mice^19^, highlighting the critical function of mPFC in regulating depression-related behavior. However, the identity and the spectrum of molecular changes in the mPFC mediating depressive-like behavior has not been fully elucidated.

Glucose is a major energy source in the brain^20^ and, notably, impaired glucose metabolism is strongly associated with major depressive disorders^20–22^. For example, in patients with depressive disorder who showed metabolic abnormalities in the prefrontal cortex region, the administration of antidepressant drugs restored the glucose metabolism^23^. While the majority of glucose is used to generate ATP, a small percentage of glucose is converted to UDP-GlcNAc via the hexosamine biosynthetic pathway (HBP)^24,25^. The UDP-GlcNAc serves as a donor molecule to transfer GlcNAc to proteins (O-GlcNAcylation), and the attachment of GlcNAc to serine or threonine residues is highly dynamic and reversible^25–28^. The addition and removal of the GlcNAc moiety are entirely mediated by O-GlcNAc transferase (OGT) and O-GlcNAcase (OGA), respectively. Both OGT and OGA are highly expressed in the brain, and particularly enriched in the cortex and hippocampus^29^. Interestingly, abnormal O-GlcNAcylation levels are closely associated with deficits in brain functions, such as synaptic plasticity and intracellular signaling^30–32^.

Recently, two research groups, including our own laboratory, have independently reported that O-GlcNAc modification in the hippocampus plays an important role in regulating neuronal excitability as well as cognitive functions^31–34^. In addition, impaired O-GlcNAc cycling is also implicated in pathological conditions, such as diabetes and Alzheimer diseases^26,35^. However, it is currently unknown whether the altered O-GlcNAcylation modulates synaptic functions involved in depressive behavior. In this study, we found that Oga heterozygous (*Oga*^+/−^) mice with chronically elevated O-GlcNAcylation exhibited antidepressant-like behaviors, and aimed to investigate the molecular mechanism underlying the behavioral effects by revealing the associated change in synaptic transmission in the mPFC.

## Results

### *Oga*^+/−^ mice exhibit antidepressant-like behaviors

**Figure 1:**
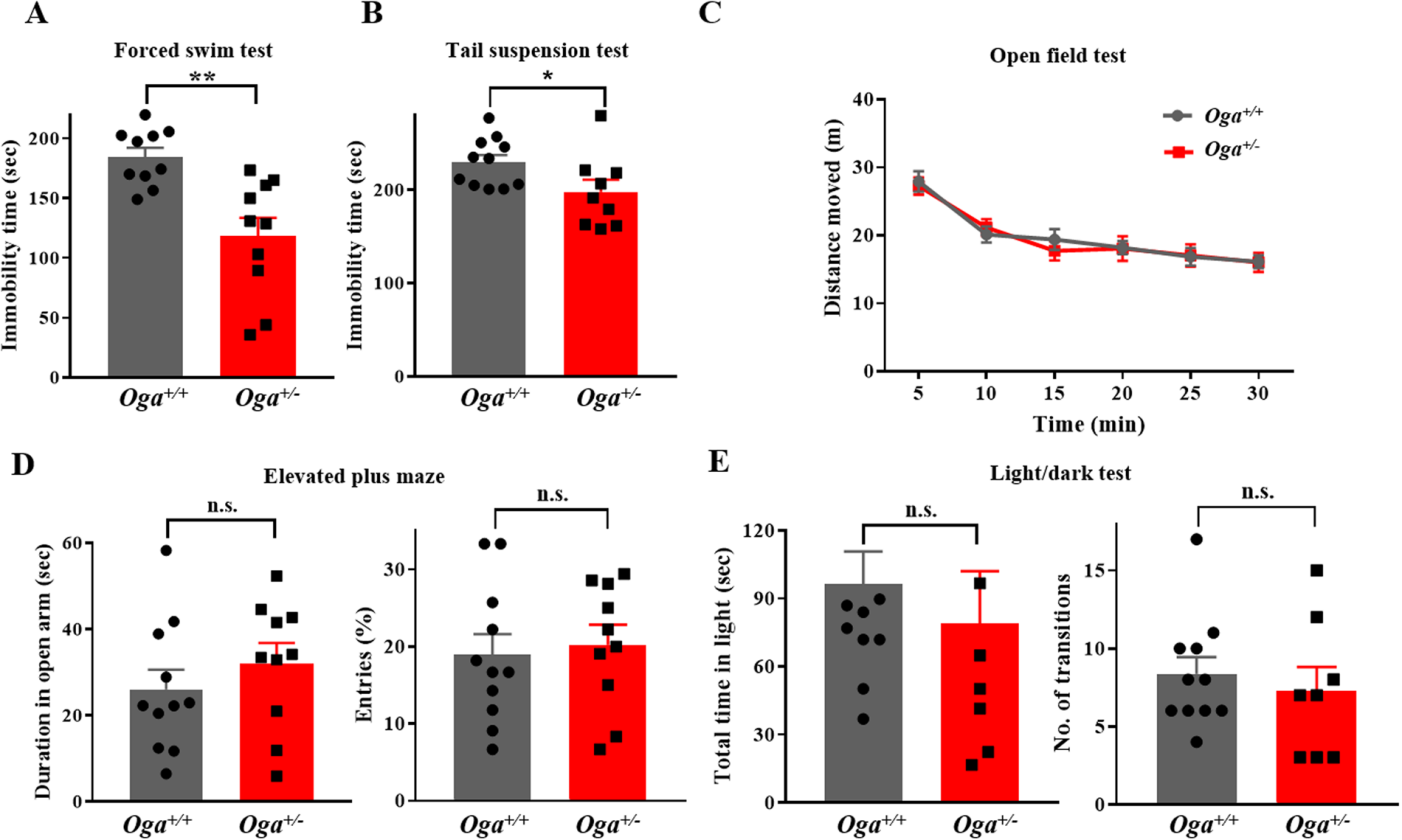
*Oga heterozygous* (*Oga*^+/−^) mice show depression-resistant behaviors. Depressive-like behavioral tests in *Oga*^+/+^ (grey bar) and *Oga*^+/−^ (red bar) mice. **(A)** Immobility time in the force swim test (*Oga*^+/+^, n = 10; *Oga*^+/−^, n = 10; t_(18)_ = 3.851, P = 0.0012). **(B)** Immobility time in the tail suspension test (*Oga*^+/+^, n = 11; *Oga*^+/−^, n = 9; t_(18)_ = 2.167, P = 0.0439). **(C)** Trace of movement (left), total distance moved (central) and distance vs time plot (right) in the open field test (*Oga*^+/+^, n = 13; *Oga*^+/−^, n = 11; t_(22)_ = 0.1727, P = 0.8645). **(D)** Duration (left; t_(19)_ = 0.9161, P = 0.3711) and percentage of entries to open arms (right; t_(19)_ = 0.3561, P = 0.7257) in the elevated plus maze test (*Oga*^+/+^, n = 11; *Oga*^+/−^, n = 10). **(E)** Total time in light box (left; t_(17)_ = 0.6685, P = 0.5128) and the number of transition between light and dark boxes (right; t_(17)_ = 0.6058, P = 0.5526) (*Oga*^+/+^, n = 11; *Oga*^+/−^, n = 8). **p < 0.01, *p < 0.05, n.s.: not significant, Student’s unpaired *t*-test.

A deficit in glucose metabolism leads to an increased risk for neuropsychiatric diseases, such as depression and anxiety, and patients with depressive disorders exhibit reduced glucose metabolic activity in the mPFC^20–23^. In addition, elevated blood glucose levels induced by a high-fat diet have been shown to modulate depression and anxiolytic-like behaviors^36^. In this study, we used O-GlcNAcase (OGA) heterozygous (*Oga*^+/−^) mice with chronically elevated O-GlcNAcylation levels, and examined whether a change in O-GlcNAcylation affects depressive-like behaviors. In the forced swim test (FST), *Oga*^+/−^ mice showed significantly reduced immobility time compared to the control (*Oga*^+/+^) group (Fig. 1A), indicating less despair behaviors upon decreased OGA levels. In tail suspension test (TST), another commonly used behavioral paradigm to measure helplessness in mice, similarly to the FST, *Oga*^+/−^ mice showed a significant reduction in immobility time compared to the *Oga*^+/+^ control mice (Fig. 1B). Locomotor activity remained unchanged in *Oga*^+/−^ mice when measured with the open field test (Fig. 1C). We also examined anxiety levels in *Oga*^+/−^ mice by using an elevated plus maze (EPM) test as well as a light/dark test, and found that basal anxiety levels were comparable between the two groups (Fig. 1D,E). Taken together, these data indicate that chronically increased O-GlcNAcylation levels lead to an antidepressant-like phenotype.

### Elevated O-GlcNAcylation levels alter synaptic transmission in the mPFC

**Figure 2:**
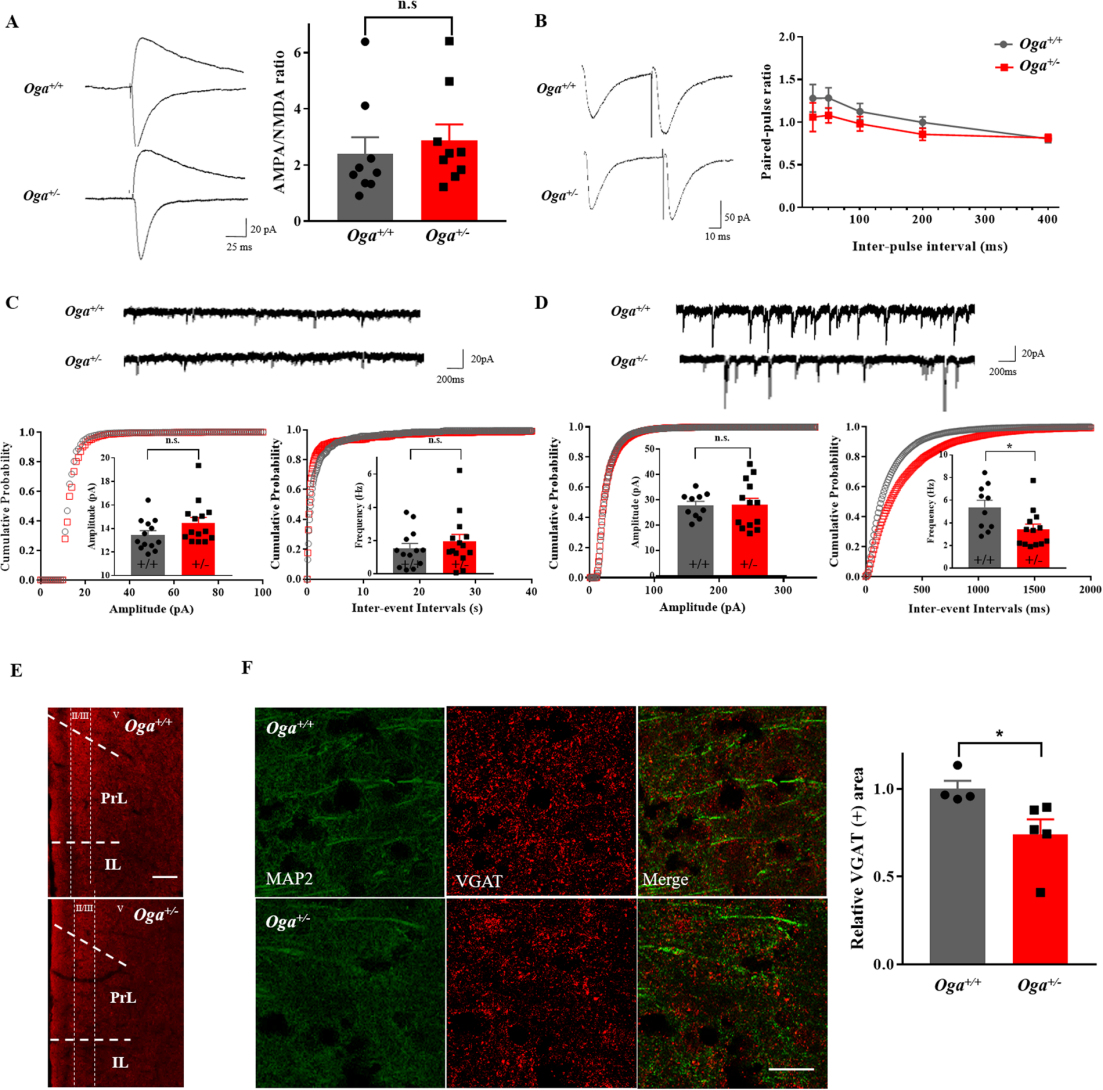
*Oga*^+/−^ mice exhibit a significant decrease in sIPSC frequency in mPFC layer II/III neurons. Recordings were made from pyramidal neurons in the mPFC layer II/III. **(A)** Representative traces of AMPA/NMDA currents (left) and averaged AMPA/NMDA ratio (right) (*Oga*^+/+^, n = 9; *Oga*^+/−^, n = 9; t_(16)_ = 0.5875, P = 0.5651). **(B)** Representative traces of paired-pulse responses (left) and averaged paired-pulse ratio with different inter-pulse intervals (right) (*Oga*^+/+^, n = 9; *Oga*^+/−^, n = 10; 25 ms t_(17)_ = 0.945, P = 0.3579; 50 ms t_(17)_= 1.84, P = 0.1842; 100 ms t_(17)_ = 1.13, P = 0.2741; 200 ms t_(17)_ = 1.457, P = 0.1633; 400 ms t_(17)_ = 0.1593, P = 0.8753). **(C)** Representative traces of sEPSCs (upper), cumulative probability plots of sEPSC amplitude (lower left; t_(25)_ = 1.635, P = 0.1145) and inter-event intervals (lower right; t_(25)_ = 0.8159, P = 0.4223) (*Oga*^+/+^, n = 13; *Oga*^+/−^, n = 14). **(D)** Representative traces of sIPSCs (upper), cumulative probability plots of sIPSC amplitude (lower left; t_(21)_ = 0.03928, P = 0.9690) and inter-event intervals (lower right; t_(21)_ = 2.561, P = 0.0182) (*Oga*^+/+^, n = 10; *Oga*^+/−^, n = 13). **(E)** Low-magnification images of VGAT staining in the mPFC areas from *Oga*^+/+^ and *Oga*^+/−^ mice. **(F)** Representative immunofluorescence images of VGAT-positive puncta in the mPFC (left, scale bar, 25 μm), and the graph of normalized VGAT-positive area (*Oga*^+/+^, N = 4, n = 15; *Oga*^+/−^, N = 5, n = 18; t_(7)_ = 2.44, P = 0.0447). N and n indicate the total number of mice and the number of mPFC slices examined, respectively. *p < 0.05, n.s.: not significant, Student’s unpaired *t*-test.

The mPFC is primarily associated with the regulation of emotional states. Given the fact that the mPFC is enriched for the expression of OGT and OGA enzymes^29^, we tested whether elevated O-GlcNAcylation levels affect neural activities in the mPFC. First, basal synaptic transmission mediated by α-amino-3-hydroxy-5-methyl-4-isoxazolepropionic acid (AMPA) and NMDA receptors were examined using whole-cell patch clamp. The AMPA/NMDA ratio measured in layer II/III neurons of the mPFC was not altered in *Oga*^+/−^ mice (Fig. 2A), indicating that excitatory synaptic transmission does not change with OGA heterozygosity in the mPFC prelimbic cortex (PrL). We also examined tentative changes in the presynaptic release probability by measuring the paired-pulse ratio (PPR), and found that the PPRs recorded at multiple inter-pulse intervals remained unchanged in *Oga*^+/−^ mice (Fig. 2B).

We next examined spontaneous excitatory postsynaptic currents (sEPSCs) and inhibitory post-synaptic currents (sIPSCs) in layer II/III neurons. Interestingly, while the amplitude and the frequency of sEPSCs were not altered in *Oga*^+/−^ mice (Fig. 2C), the sIPSC frequency was significantly decreased in *Oga*^+/−^ mice (Fig. 2D, right) without any change in the sIPSC amplitude (Fig. 2D, left). When measuring the sIPSCs in three other layers of the mPFC (PrL layer V, infralimbic cortex layer II/III, and layer V), neither the amplitude nor the frequency of sIPSCs were changed in *Oga*^+/−^ mice (Supplementary Fig. S1), in contrast to the changes observed in PrL layer II/III neurons. Miniature excitatory postsynaptic currents (mEPSCs) and inhibitory postsynaptic currents (mIPSCs) were also recorded in PrL layer II/III neurons, and only the frequency of mIPSCs was reduced, which is in agreement with the sEPSC and sIPSC recordings (Supplementary Fig. S2). These results suggest that O-GlcNAcylation levels shift the GABAergic and glutamatergic balance by modulating basal inhibitory GABAergic synaptic transmission in PrL layer II/III neurons.

Given the changes in the frequency of sIPSCs and mIPSCs in *Oga*^+/−^ mice, we measured the density of synaptic vesicle proteins by immunostaining with either vesicular GABA transporter (VGAT) or vesicular glutamate transporter (VGluT) antibody in the PrL layer II/III. Consistent with the decreased sIPSC and mIPSC frequency in *Oga*^+/−^ mice, the area of VGAT-positive puncta was significantly diminished in the PrL layer II/III of *Oga*^+/−^ mice compared to *Oga*^+/+^ control mice (Fig. 2E,F). In contrast, the area of VGAT-positive puncta was not significantly different in PrL layer V, IL layer II/III and IL layer V between *Oga*^+/+^ and *Oga*^+/−^ mice (Supplementary Fig. S3), indicating that the reduction in VGAT-positive area is specific to PrL layer II/III. The amount of inhibitory synapses was also quantified using gephyrin, a postsynaptic marker of inhibitory synapses, and similarly to the case of VGAT, the area of gephyrin-positive punctas were reduced in the PrL layer II/III of *Oga*^+/−^ mice (Supplementary Fig. S4), while the area of VGlut1-positive puncta was not affected in PrL layer II/III (Supplementary Fig. S5). These results indicate that the decrease in the number of inhibitory synapses contributed to the reduced frequency of inhibitory synaptic transmission in *Oga*^+/−^ mice.

### OGA overexpression rescues the reduced sIPSC frequency in the mPFC

**Figure 3:**
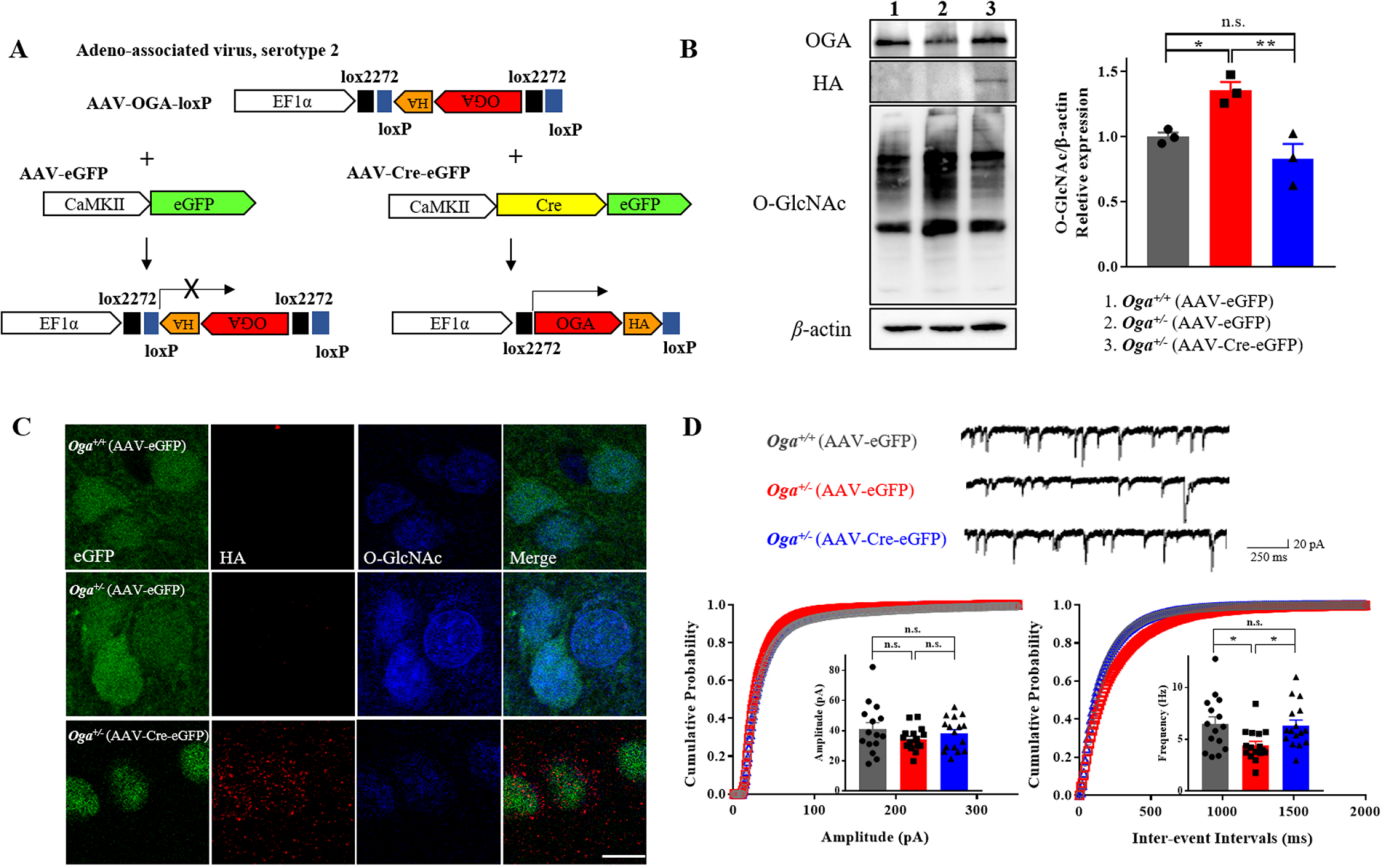
OGA overexpression in the mPFC rescues a decrease in sIPSC frequency in *Oga*^+/−^ mice. **(A)** Schematic representation of Cre-loxP recombination system for viral-mediated gene overexpression. **(B)** Immunoblot analysis of OGA expression (upper) and overall levels of O-GlcNAcylated proteins (left), and the graph of normalized O-GlcNAcylation levels (right; F_(2,6)_ = 11.81, P = 0.0083) (AAV-eGFP in *Oga*^+^/^+^, n = 3; AAV-eGFP in *Oga*^+/−^, n = 3, AAV-Cre-eGFP in *Oga*^+/−^, n = 3). **(C)** Validation of OGA-HA expression in PrL layer II/III neurons. Representative immunofluorescence images of HA-tag expression (red) and O-GlcNAcylation levels (blue) in the mPFC are shown. Cre-eGFP includes nuclear localization signal, thus its expression is restricted within the nucleus (scale bar, 10 μm). **(D)** Representative traces of sIPSCs recorded from the PrL layer II/III neurons (upper). Cumulative probability plots of sIPSC amplitude (lower left; F_(2,44)_ = 1.156, P = 0.3241) and inter-event intervals (lower right; F_(2,44)_ = 4.586, P = 0.0155) (AAV-eGFP in *Oga*^+^/^+^, n = 15; AAV-eGFP in *Oga*^+/−^, n = 16, AAV-Cre-eGFP in *Oga*^+/−^, n = 16). *p < 0.05, n.s.: not significant, one-way ANOVA followed by Tukey’s multiple comparisons *post hoc* test. Full-length blots are presented in the Supplementary Fig. S13.

Given the decrease in sIPSC frequency in *Oga*^+/−^ mice (Fig. 2D), we examined whether restoring OGA levels in PrL layer II/III neurons rescues the reduced sIPSC frequency. We used adeno-associated viruses (AAVs) expressing Cre-eGFP along with OGA-loxP (AAV-EFα1-loxP-OGA-HA-loxP) as illustrated in Fig. 3A. The expression levels of OGA and O-GlcNAcylated proteins in the mPFC were examined in control and virus-injected mice using western blotting. The *Oga*^+/−^ mice injected with Cre-eGFP/OGA-loxP showed restored OGA expression as well as diminished levels of O-GlcNAcylated proteins, comparable to *Oga*^+/+^ mice (Fig. 3B). In addition to the western blot, the rescue of decreased O-GlcNAcylation levels by OGA overexpression was also validated by determining O-GlcNAcylation levels in GFP-positive neurons in PrL layer II/III (Supplementary Fig. S10C). The injection site in the PrL area of the mPFC was visually confirmed (Supplementary Fig. S6A), and the electrophysiology recording was performed 14–18 days after the AAV injection (Supplementary Fig. S6B). The specific expression of exogenous OGA tagged with HA was also confirmed by anti-HA antibody in western blot (Fig. 3B) and visualized by immunostaining in the mPFC (Fig. 3C). The co-localization of GFP with CaMKII was also confirmed by immunostaining, demonstrating the specificity of the cre-virus (Supplementary Fig. S7). When injected with the control eGFP and OGA-loxP viruses, *Oga*^+/−^ mice showed decreased sIPSC frequency (Fig. 3D, bottom right) without a change in the sIPSC amplitude (Fig. 3D, bottom left), similarly to Fig. 2D. Interestingly, when OGA was overexpressed in the mPFC PrL with the injection of Cre-eGFP/OGA-loxP in *Oga*^+/−^ mice, the reduced sIPSC frequency was rescued to control levels without affecting the sIPSC amplitude (Fig. 3D). Besides, the overexpression of OGA in *Oga*^+/−^ mice did not affect sEPSC and mEPSC in the PrL layer II/III (Supplementary Fig. S8A,B). However, similar to the sIPSC recording, mIPSC frequency showed a decreasing trend in the *Oga*^+/−^ condition, which was rescued to a control level by OGA overexpression (Supplementary Fig. S8C). Moreover, the reduction in the area of VGAT-positive puncta observed in *Oga*^+/−^ mice (Fig. 2F) was also rescued by the overexpression of OGA (Supplementary Fig. S9). These results suggest that the degree of O-GlcNAcylation specifically modulates the frequency of inhibitory synaptic transmission in PrL layer II/III neurons of the mPFC.

### OGA overexpression in the mPFC affects antidepressant-like behaviors *in vivo*

**Figure 4:**
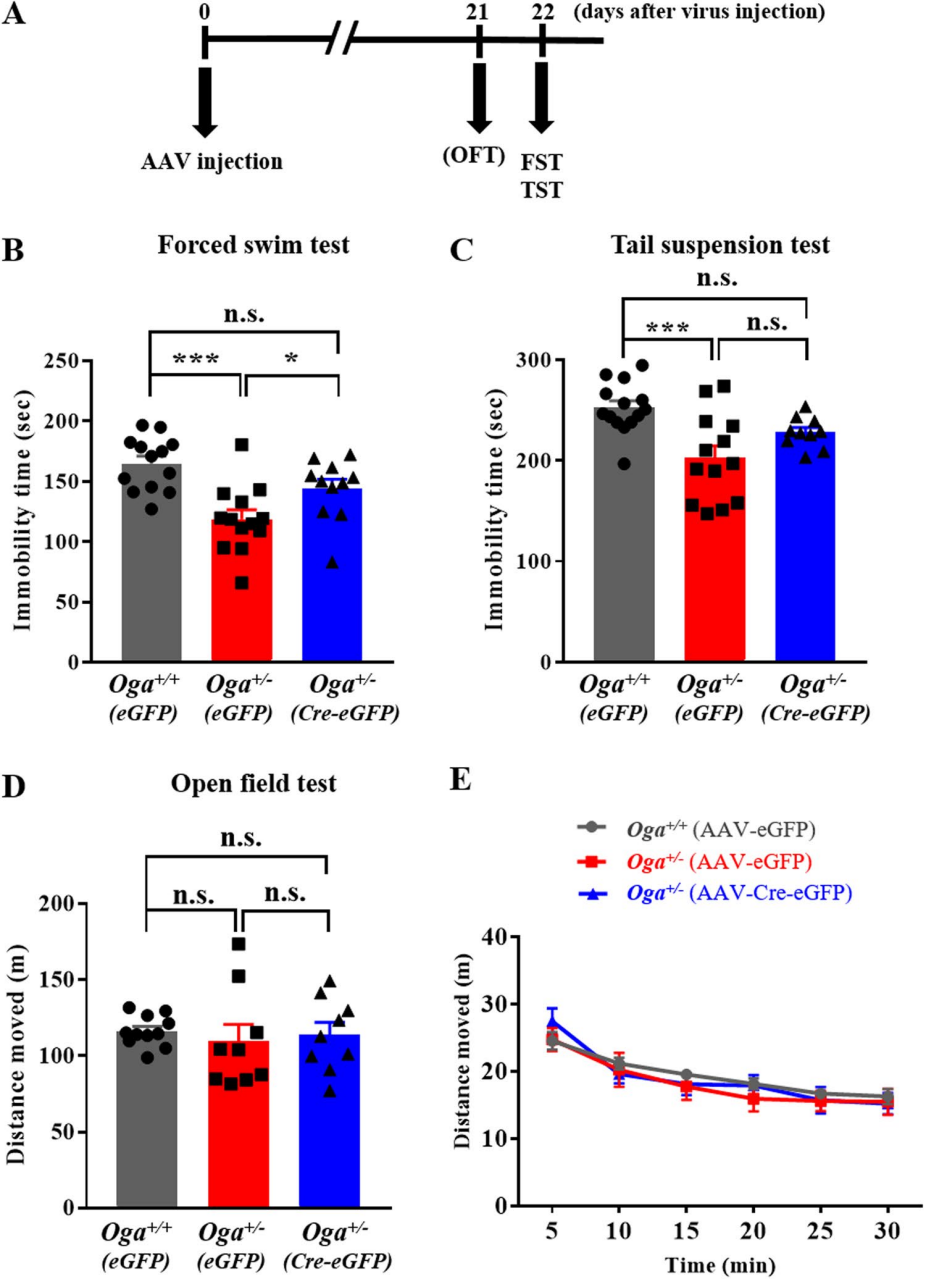
OGA overexpression in the mPFC inhibits depression-resistant behaviors in *Oga*^+/−^ mice. **(A)** Experimental timeline for behavioral experiments after virus injection. **(B)** Forced swim test in *Oga*^+/+^ or *Oga*^+/−^ mice with the expression of AAV-eGFP or AAV-Cre-eGFP in the mPFC (AAV-eGFP in *Oga*^+^/^+^, n = 13; AAV-eGFP in *Oga*^+/−^, n = 13, AAV-Cre-eGFP in *Oga*^+/−^, n = 11; F_(2, 34)_ = 10.83, P = 0.0002). **(C)** Tail suspension test in *Oga*^+^/^+^ or *Oga*^+/−^ mice with expression of AAV-eGFP or AAV-Cre-eGFP in the mPFC (AAV-eGFP in *Oga*^+^/^+^, n = 14; AAV-eGFP in *Oga*^+/−^, n = 13, AAV-Cre-eGFP in *Oga*^+/−^, n = 10; F_(2, 34)_ = 8.816, P = 0.0008). **(D)**, **(E)** Open field test in *Oga*^+^/^+^ or *Oga*^+/−^ mice with expression of AAV-eGFP or AAV-Cre-eGFP in the mPFC (AAV-eGFP in *Oga*^+^/^+^, n = 11; AAV-eGFP in *Oga*^+/−^, n = 9, AAV-Cre-eGFP in *Oga*^+/−^, n = 9; F_(2, 26)_ = 0.2007, P = 0.8194). ***p < 0.001, **p < 0.01, *p < 0.05, n.s.: not significant, one-way ANOVA followed by Tukey’s multiple comparisons *post hoc* test.

Considering that the reduction in sIPSC frequency was rescued with OGA overexpression in *Oga*^+/−^ mice (Fig. 3D), we further examined whether OGA overexpression mediates antidepressant-like behaviors using the FST and TST. Open field test was performed 21 days after the injection of AAV via stereotaxic surgery, and FST or TST was performed the next day (Fig. 4A). Consistent with the experiments without viral injection (Fig. 1A,B), *Oga*^+/−^ mice injected with a control eGFP virus showed significantly reduced immobility time in both the FST and the TST, compared to the *Oga*^+/+^ mice injected with a control virus (Fig. 4B,C). When injected with the Cre-eGFP virus, the reduced immobility time in the FST was restored to control levels in *Oga*^+/−^ mice (Fig. 4B). The immobility time in the TST also showed an increasing trend in *Oga*^+/−^ mice when OGA was overexpressed in the mPFC (Fig. 4C). The locomotor activities measured using the open field test were not affected by the injection of a control eGFP or Cre-eGFP virus along with the OGA-loxP virus (Fig. 4D,E). These results together indicate that O-GlcNAcylation levels in the mPFC modulate antidepressant-like behaviors.

## Discussion

Previous studies demonstrated that both spatial learning and hippocampal synaptic plasticity are impaired in *Oga*^+/−^ mice^31,32^, and that a deficit in O-GlcNAc cycling is associated with neurodegenerative and metabolic diseases^37,38^. The immediate donor molecule for O-GlcNAcylation, UDP-GlcNAc, is produced from glucose via the HBP, and thus, the degree of O-GlcNAc modification changes rapidly depending on blood glucose levels. O-GlcNAcylation also influences metabolic homeostasis^39^ and, interestingly, the glucose metabolism is impaired in the brain of patients with depression^21,23^. However, it was not clear whether O-GlcNAc modification directly modulates depression-related behaviors. In this study, using *Oga*^+/−^ mice with chronically elevated O-GlcNAcylation levels, we found that *Oga*^+/−^ mice exhibited an antidepressant-like phenotype, and that sIPSC frequency was reduced in the PrL layer II/III of the mPFC. Both the antidepressant-like phenotype and changes in sIPSC frequency in PrL layer II/III neurons were restored by the overexpression of OGA, suggesting that the regulation of GABAergic transmission through O-GlcNAcylation in the mPFC modulates antidepressant-like behaviors. Altered GABAergic transmission and dysregulated function of GABA_A_ receptors are observed in patients with major depressive disorder^9^, and the synaptic function in the mPFC is closely linked to antidepressant-like behaviors^19^. A diverse population of GABAergic inhibitory neurons exist in the brain with distinct biochemical, electrophysiological and morphological features, such as parvalbumin (PV)-, calretinin (CR)-, calbindin (CB)-, cholecystokinin (CCK)-, somatostatin (SOM)- and neuropeptide Y (NPY)-positive GABAergic neurons^40^. CCK- and PV-positive GABAergic neurons are highly abundant in the mPFC^41^, and among them, CCK-positive GABAergic neurons were particularly vulnerable to stress in the PrL region^42^. The vulnerability of SOM-positive interneurons to stress has also been demonstrated^9,43^, implying the regulation of dendritic excitability of PrL layer II/III neurons by SOM interneurons possibly contributes to the antidepressant-like behavior in *Oga*^+/−^ mice. However, a further study with systematic molecular manipulation is necessary to reveal the identity of the subpopulation of GABAergic neurons involved in the modulation of an antidepressant-like phenotype through O-GlcNAcylation.

In contrast to a decrease in sIPSC and mIPSC frequency in PrL layer II/III neurons, our group previously reported that mIPSC frequency remains unchanged in hippocampal CA1 neurons in *Oga*^+/−^ mice^31^, indicating that the PrL layer II/III neurons are more sensitive to a decrease in OGA expression levels. This difference may result from the fact that the hippocampus is enriched with particularly high levels of OGT and OGA transcripts^44^, which may help to cope with the OGA heterozygosity. Moreover, it is also intriguing that the reduction in sIPSC frequency was only observed in PrL layer II/III neurons, but not in PrL layer V, IL layer II/III, and IL layer V of the mPFC. This difference presumably results from the structurally and functionally distinct nature of neurons in the PrL and IL subregions^45–47^, and the functional significance of PrL layer II/III neurons in regulating depressive-like phenotypes has been reported previously^15,17,19^. In particular, pyramidal neurons exhibited distinct electrophysiological and/or morphological properties, and a different susceptibility to spared nerve injury across different subregions of the mPFC^47^. A potential difference in the subpopulation of GABAergic inhibitory neurons as well as local inhibitory circuits across the mPFC subregions may also contribute to the specific changes in PrL layer II/III neurons^48^. Importantly, it was previously unknown whether the levels of O-GlcNAcylation are different across the mPFC subregions, and we found that O-GlcNAclyation levels were particularly enriched in PrL layer II/III compared to other three regions (PrL layer V, IL layer II/III and IL layer V) in *Oga*^+/+^ mice (Supplementary Fig. S10A). In contrast, O-GlcNAcylation levels in PrL layer II/III were not significantly higher compared to other three regions in *Oga*^+/−^ mice (Supplementary Fig. S10B). Given that a change in inhibitory synaptic transmission was only observed in the PrL layer II/III, it is possible that the particularly high levels of O-GlcNAcylation in PrL layer II/III neurons make them more vulnerable to Oga heterozygosity.

In the Cre-loxP system used to rescue OGA levels in the mPFC, Cre was expressed in excitatory neurons under the CaMKII promoter, suggesting that the reduction in the inhibitory synaptic transmission observed in *Oga*^+/−^ mice was mediated by a change in the postsynaptic compartment. Several postsynaptic components are implicated in the GABAergic synaptic transmission, such as neuroligin 2 and Slitrk3^49–51^. A recent advance in proteomic techniques enabled the identification of numerous neuronal proteins and ion channels undergoing O-GlcNAcylation^52,53^, and neuroligin 2 is heavily modified by diverse PTMs^54^. Given that O-GlcNAc modification influences diverse aspects of protein functions, including protein-protein interaction or localization^37^, a shift in O-GlcNAcylation levels may affect the scaffolding or adhesion proteins involved in the maintenance of inhibitory synapses in the postsynaptic compartment. Immunostaining with VGAT confirmed the reduction in the number of inhibitory synapses, and therefore, it is likely that O-GlcNAcylation of postsynaptic scaffolding proteins contributes to the maintenance of inhibitory synapses in the mPFC. However, it remains to be examined whether postsynaptic proteins involved in the maintenance of GABAergic transmission are directly modified by O-GlcNAcylation.

The mPFC is involved in the top-down neural control of stress-response adaptation, and elevated mPFC neuronal activity has been shown to suppress stress-mediated activity of the hypothalamic-pituitary-adrenal (HPA) axis implicated in depression^55^. Therefore, the decreased GABAergic transmission in PrL layer II/III neurons is expected to attenuate HPA-axis activity and the release of glucocorticoid (GC) hormones in *Oga*^+/−^ mice. Interestingly, a recent finding suggested that the HPA-axis is also impacted by O-GlcNAc modification^38^, and therefore elevated O-GlcNAcylation levels in *Oga*^+/−^ mice may directly affect an antidepressant-like phenotype via the HPA-axis.

Given the close association between a chronic increase in O-GlcNAcylation levels in *Oga*^+/−^ mice and an antidepressant-like phenotype, we also examined whether the administration of an antidepressant drug influences the degree of O-GlcNAcylation in the mPFC. Based on a previously published study^56^, fluoxetine was intraperitoneally injected (20 mg/kg), and the acute effect of fluoxetine administration on O-GlcNAcylation levels was examined 30 min after the injection. Interestingly, fluoxetine administration significantly decreased the levels of O-GlcNAcylation in the mPFC (Supplementary Fig. S11), which is in contrast to the antidepressant-like phenotype we observed in *Oga*^+/−^ mice with chronically elevated O-GlcNAcylation levels. This discrepancy may result from the fact the failure to maintain an adequate range of intracellular O-GlcNAcylation levels can lead to abnormal cellular responses, as proposed in a review by Yang and Qian^37^. For example, in the hippocampus, both increasing and decreasing O-GlcNAcylation levels caused a deficit in synaptic plasticity rather than modulating synaptic plasticity in a bidirectional manner^24^. It is also possible that an acute change in O-GlcNAcylation levels modulates an antidepressant-like phenotype via a different mechanism compared to a chronic change in O-GlcNAcylation levels. Further study is necessary to address a potential convergence of between the molecular action of antidepressant drugs and O-GlcNAc-mediated signaling pathways. It would be also interesting to examine other classes of antidepressant drugs, and their acute versus chronic effects on O-GlcNAcylation levels.

In conclusion, our study demonstrates that chronically elevated O-GlcNAcylation is associated with an antidepressant-like phenotype, and that the regulation of O-GlcNAcylation levels in the mPFC plays a crucial role in maintaining inhibitory synaptic transmission as well as in mediating antidepressant-like behaviors. Moreover, the close association observed between O-GlcNAcylation levels and the antidepressant-like phenotype suggests the modulation of O-GlcNAcylation levels as a novel target for antidepressant therapies.

## Materials and Methods

### Animals

*Oga*^+/−^ mice in C57BL/6J background were generated previously^57^. All mice were maintained with free access to food and water under a 12-hour light/dark cycle. Animal care and experimental procedures adhered to the Guide for the Care and Use of Laboratory Animals and were approved by the Institutional Animal Care and Use Committee of the Korea Institute of Science and Technology (KIST).

### Behavioral tests

All mice (8–9 weeks) were habituated to the behavioral testing room for a minimum of 30 min before the start of each behavioral task, and white noise (65–70 dB) was continuously present to mask extraneous sound. Behavioral tests and locomotor activity were videotaped and the data were analyzed using EthoVision 9.0 software (Noldus Information Technology, Wageningen, Netherlands). Anxiety tests were analyzed manually via video monitoring. The open field test, elevated plus maze, light/dark box test were performed in the same batch of mice. For the forced swim test and tail suspension test, a new and dedicated batch of mice were used for each experiment. Control mice (*OGA*^+/+^) were littermates of the *OGA*^+/−^ mice for all experiments.

#### Forced swim test

Mice were placed in a clear plastic cylinder (25-cm height, 15-cm diameter) containing water with a depth of 15 cm at 23–25 °C. Each test session lasted for 6 min; the first 2 min for habituation and the next 4 min for behavioral monitoring. After the test, mice were dried with paper towel and returned to home cage. The immobility time in each mouse was analyzed.

#### Tail suspension test

Mice were individually suspended by their tails to a ring-shaped bar on the top of a box (33 ×33 ×45 cm). The tip of the tail was fixed using paper tape, and immobility time was measured for a duration of 6 min. Mice that managed to climb above the ring-shaped bar were excluded from data analysis.

#### Open field test

The open field test was performed in an open field apparatus (40 ×40 cm). Mice were placed in the open field box and locomotor activities were monitored for 30 min. Total distance and the distance moved for every 5 min window were analyzed using the Ethovision software.

#### Elevated plus maze

The elevated plus maze consisted of two open arms (25 ×8 cm), two closed arms (25 ×8 cm) and a central square platform (8 ×8 cm) raised 50 cm above the floor. Mice were placed in the platform facing a closed arm. Durations and the number of entries into the open and closed arms were recorded for 5 min.

#### Light/dark test

The apparatus consisted of two separate compartments (25 × 40 × 20 cm). The light compartment was open and exposed to bright light (600 lux), while the dark compartment was covered and kept dark at all times. Each mouse was placed in the dark box and allowed to move between the light and dark fields. Total time in the light box and the number of transitions were recorded for 10 min.

### mPFC slice preparation

4- to 5-week-old male *Oga*^+/+^ and *Oga*^+/−^ mice were anesthetized with halothane. After confirming a state of proper anesthesia, mPFC slices (300-μm thickness) from each mouse were prepared using a vibratome (Leica VT1000S; Leica, Nussloch, Germany) in an ice-cold buffer containing (in mM) 234 sucrose, 2.5 KCl, 1.25 NaH_2_PO_4_, 24 NaHCO_3_, 10 glucose, 0.5 CaCl_2_, and 10 MgSO_4_, and constantly bubbled with 95% O_2_ and 5% CO_2_. The slices were recovered at 35 °C for one hour in a recovery artificial cerebrospinal fluid (aCSF) solution and maintained at room temperature until the beginning of each experiment. The slices were then transferred to a submerged recording chamber and were continuously perfused with the recording aCSF solution (in mM: 119 NaCl, 2.5 KCl, 1.25 NaH_2_PO_4_, 26 NaHCO_3_, 10 glucose, 2.5 CaCl_2_, 2 MgSO_4_), bubbled with 95% O_2_ and 5% CO_2_.

### Electrophysiology

Spontaneous excitatory postsynaptic currents (sEPSCs) were recorded in the recording aCSF supplemented with 50 μM D-APV and 50 μM picrotoxin. For recording miniature excitatory postsynaptic currents (mEPSCs), aCSF was supplemented with 1 μM TTX, 50 μM D-APV, and 50 μM picrotoxin. For whole-cell recordings of mPFC layer II/III neurons in a voltage-clamp configuration, glass pipettes of 3–5 MΩ resistance were used. The cells were held at −70 mV, and the internal solution contained (in mM) 125 CsMeSO_3_, 2.8 NaCl, 20 HEPES, 0.4 EGTA, 4 ATP-Mg, 0.5 GTP-Na_2_, 10 phosphocreatine-Na_2,_ 5 QX-314 (pH = 7.25 and osmolality = 285–290 mOsm). To record inhibitory postsynaptic currents, aCSF was supplemented with 50 μM D-APV and 10 μM DNQX for spontaneous inhibitory postsynaptic currents (sIPSCs) or with 1 μM TTX, 50 μM D-APV, and 10 μM DNQX for miniature inhibitory postsynaptic currents (mIPSCs). The cells were held at −70 mV, and the internal solution contained (in mM) 134 CsCl, 2 MgCl_2_, 10 HEPES, 1 EGTA, 2ATP-Mg, 0.5 GTP-Na_2_, 5 phosphocreatine-Na_2_ (pH = 7.25 and osmolality = 285–290 mOsm). For recording evoked EPSCs (eEPSCs) and the paired pulse ratio (PPR), cesium-based intracellular solution (125 CsMeSO_3_, 2.8 NaCl, 20 HEPES, 0.4 EGTA, 4 ATP-Mg, 0.5 GTP-Na_2_, 10 phosphocreatine-Na_2_, 5 QX-314) was used. The stimulating electrode was placed 50–100 μm lateral to the recording site in layer II/III. The AMPA/NMDA ratio was recorded at −70 and +40 mV holding potentials. Each response was recorded for 20 sweeps with 20-sec intervals. To measure the PPR, paired stimulations with inter-stimulus intervals of 25, 50, 100, 200, and 400 ms were applied. Each response was recorded for 10 sweeps with 20-sec intervals.

### Viruses

A Cre/loxP recombination system was used for the overexpression of OGA in mPFC neurons. AAV1.CamKII0.4.eGFP.WPRE.rBG (AAV-eGFP) and CamKII.HI.eGFP-Cre.WPRE.SV40 (AAV-eGFP-Cre) viruses were purchased from the University of Pennsylvania Vector Core (Philadelphia, PA, USA). For Cre-mediated inversion of the flanked OGA, AAV-EFα1-loxP-OGA-HA-loxP (AAV-OGA-loxP) virus was purchased from the KIST Virus Facility (Seoul, South Korea).

### Stereotaxic surgery

Mice were anesthetized with isoflurane and placed on a stereotaxic frame (Neurostar, Tübingen, Germany) for viral injection. Two AAV mixtures were injected into the mPFC for gene overexpression; AAV1.CamKII0.4.eGFP.WPRE.rBG (serotype 2.1, ddTiter: 6.028e13 GC/ml) and AAV-EFα1-loxP-OGA-HA-loxP (serotype 2, ddTiter: 8.59e12 GC/ml) mixture was used for the control. 0.2 μL of the mixture was injected to a targeted site for control condition. CamKII.HI.eGFP-Cre.WPRE.SV40 (serotype 2, ddTiter: 1.76e13 GC/ml) and AAV-EFα1-loxP-OGA-HA-loxP mixture was used for the experimental group. 0.45 μL of the mixture was injected to a targeted site for the overexpression condition. Virus was delivered into the prelimbic mPFC (AP: + 2.0 mm ML: ± 0.3 mm DV: −2.3 mm) using a 10-mL Hamilton syringe fitted with a 33-gauge needle (World Precision Instruments, Sarasota, FL, USA). Mice were subsequently returned to a home cage to recover, and used for electrophysiology, immunohistochemistry or behavioral experiments 2–3 weeks after the surgery.

### Immunohistochemistry

40-μm coronal brain sections from each mouse were prepared and rinsed with PBS three times. After blocking with 3% normal goat serum, 0.3% triton X-100 in PBS, neuronal cells were labeled with anti-MAP2 (Cat #: ab5392, Abcam, 1:2000) antibody. VGAT and VGlut1 were labeled with anti-VGAT antibody (Cat #: 131002, Synaptic Systems, 1:300) and anti-VGlut1 antibody (Cat #: 135303, Synaptic Systems, 1:800), respectively. HA-tagged OGA was labeled with anti-HA-Tag antibody (Cat #: 3724, Cell signaling, 1:500), and O-GlcNAc-modified proteins were labeled with anti-O-GlcNAc antibody (RL2) (Cat #: MA1-072, ThermoFisher, 1:1000), anti-CaMKII antibody (Cat #: ab22609, Abcam, 1:500), anti-gephyrin (Cat #: 147011, Synaptic Systems, 1:400). After the washing, anti-rabbit Alexa-594 conjugated IgG (Cat #: ab150080, Abcam, 1:400), anti-rabbit Alexa-488 conjugated IgG (Cat #: ab150077, Abcam, 1:400) or anti-chicken Alexa-647 conjugated IgG (Cat #: ab150171, Abcam, 1:400), anti-chicken Alexa-488 conjugated IgY (Cat #: ab150173, Abcam, 1:400), anti-mouse Alexa-405 conjugated IgG (Cat #: A-31553, ThermoFisher, 1:400), anti-rabbit Alexa-647 conjugated IgG (Cat #: A-21245, ThermoFisher, 1:400) antibodies were used to visualize the signals. All slices were examined using confocal microscopy (60x objective, Olympus FV 1000, Olympus, Japan; 63x objective, Leica Application Suite X, Leica, Nussloch, Germany). VGAT- and VGlut1-positive areas in mPFC PrL layer II/III were quantified using Fiji (National Institute of Health, Bethesda, MD, USA) software. For the analysis of VGAT- and VGluT1-positive puncta in WT and *Oga*^+/−^ mice, 4–6 mice were examined per genotype. 2–4 slices were examined for each animal. Using the area selection tool of the Fiji software, VGAT- or VGluT1-positive area was measured within the region of interests (ROI) covering the PrL layer II/III. The threshold was adjusted equally across experimental conditions for the detection of VGAT- and VGluT1-positive area. In case of experiments involving AAV injection, VGAT- and VGluT1-positive areas were analyzed from 3–4 mice per condition. ROIs were randomly selected around the GFP-positive neurons in PrL layer II/III. The VGAT- or VGluT1-positive area was calculated as the ratio of VGAT- or VGluT1-positve area normalized to the total ROI area, respectively. Raw confocal images of VGAT staining used for the quantification of VGAT expression are appended in the Supplementary Fig. S12.

### Brain tissue extraction

The acute effect of antidepressant administration on O-GlcNAcylation levels in the mPFC was examined by intraperitoneal injection of fluoxetine (20 mg/kg; Sigma, St. Louis, MO, USA). 30 min after the injection, mice were sacrificed and the brain samples containing the mPFC were quickly dissected out using a stainless steel coronal brain matrix for mice. For the characterization of AAV, mice were injected with control GFP or Cre-GFP virus along with OGA-loxP via stereotaxic surgery. Two weeks after the injection, mice were sacrificed and the GFP-positive regions in the mPFC slices were dissected out. Brain tissue samples were placed on ice for 30 min, and centrifuged 13,000 rpm 20 min at 4 °C. Proteins were extracted from brain tissue samples using radioimmunoprecipitation assay (RIPA) buffer (ELPIS-BIOTECH. Inc., Daejeon, Korea), and subsequently analyzed using immunoblot.

### Western blot

For immunoblotting, tissue samples were obtained from each mouse, and protein concentrations were measured with the Bradford assay. Proteins were loaded, separated by a 10% SDS-polyacrylamide gel, and then transferred to a PVDF membrane. The membrane was blocked with Tris-buffered saline with 0.1% Tween 20 (TBST) containing either 0.5% skim milk or 0.5% BSA for one hour at room temperature. After blocking, membranes were incubated with primary antibodies, anti-MGEA5 (Cat #: 14711-1-AP, Proteintech, 1:1000), anti-O-GlcNAc (RL2) (Cat #: MA1-072, ThermoFisher, 1:1000), anti-HA-Tag (Cat #: 3724, Cell Signaling, 1:1000), or anti-*β*-actin (Cat #: Novus Biologicals, Novus, 1:50000) overnight at 4 °C. After washing six times with TBST, the membrane was incubated with secondary antibodies, anti-rabbit IgG-HRP (Cat #: SC-2004, Santa Cruz, 1:5000) or anti-mouse IgG-HRP (Cat #: 62-6520, ThermoFisher, 1:5000) for one hour at room temperature. Signals were visualized using an enhanced chemiluminescence (ECL) detection kit (Millipore, Burlington, MA, USA).

### Statistical analysis

Two group comparisons were performed using Student’s *t*-tests, and multiple comparisons were performed using one-way analysis of variance (ANOVA) followed by Tukey’s multiple comparisons *post hoc* test. All data were analyzed using GraphPad Prism 7 software (GraphPad Software Inc., La Jolla, San Jose, CA, USA) and presented as the mean ± SEM. Results were considered to be statistically significant at *p < 0.05, **p < 0.01 and ***p < 0.001.

## Supplementary information

Supplementary Information.
