# Supplementary material for: Elevated O-GlcNAcylation induces an antidepressant-like phenotype and decreased inhibitory transmission in medial prefrontal cortex
Source: Sci Rep. 2020 Apr 24;10:6924. doi: 10.1038/s41598-020-63819-6 (PMC7181662; doi:10.1038/s41598-020-63819-6)
Supplement: Supplementary file 1 — Supplementary Information. [file 41598_2020_63819_MOESM1_ESM.pdf]

Supplementary Information

Elevated O-GlcNAcylation induces an antidepressant-like phenotype and decreased inhibitory transmission in medial prefrontal cortex

Yoonjeong Cho[1,2,#], Hongik Hwang[1,#], Md. Ataur Rahman[1], Chihye Chung[3,*], and Hyewhon Rhim[1,2,*]

[1]Center for Neuroscience, Brain Science Institute, Korea Institute of Science and Technology (KIST), Seoul 02792, Republic of Korea

[2]Division of Bio-Medical Science & Technology, KIST School, Korea University of Science and Technology (UST), Seoul 02792, Republic of Korea

[3]Department of Biological Science, Konkuk University, Seoul 05029, Republic of Korea.

[#]These authors contributed equally to this work.

*Correspondence should be addressed to C.C. (cchung@konkuk.ac.kr) or H.R. (hrhim@kist.re.kr).

Corresponding Author: Hyewhon Rhim
Center for Neuroscience, Brain Science Institute
Korea Institute of Science and Technology (KIST)
Seoul 02792, Republic of Korea
(Tel.) +82-2-958-5923
(Email) hrhim@kist.re.kr

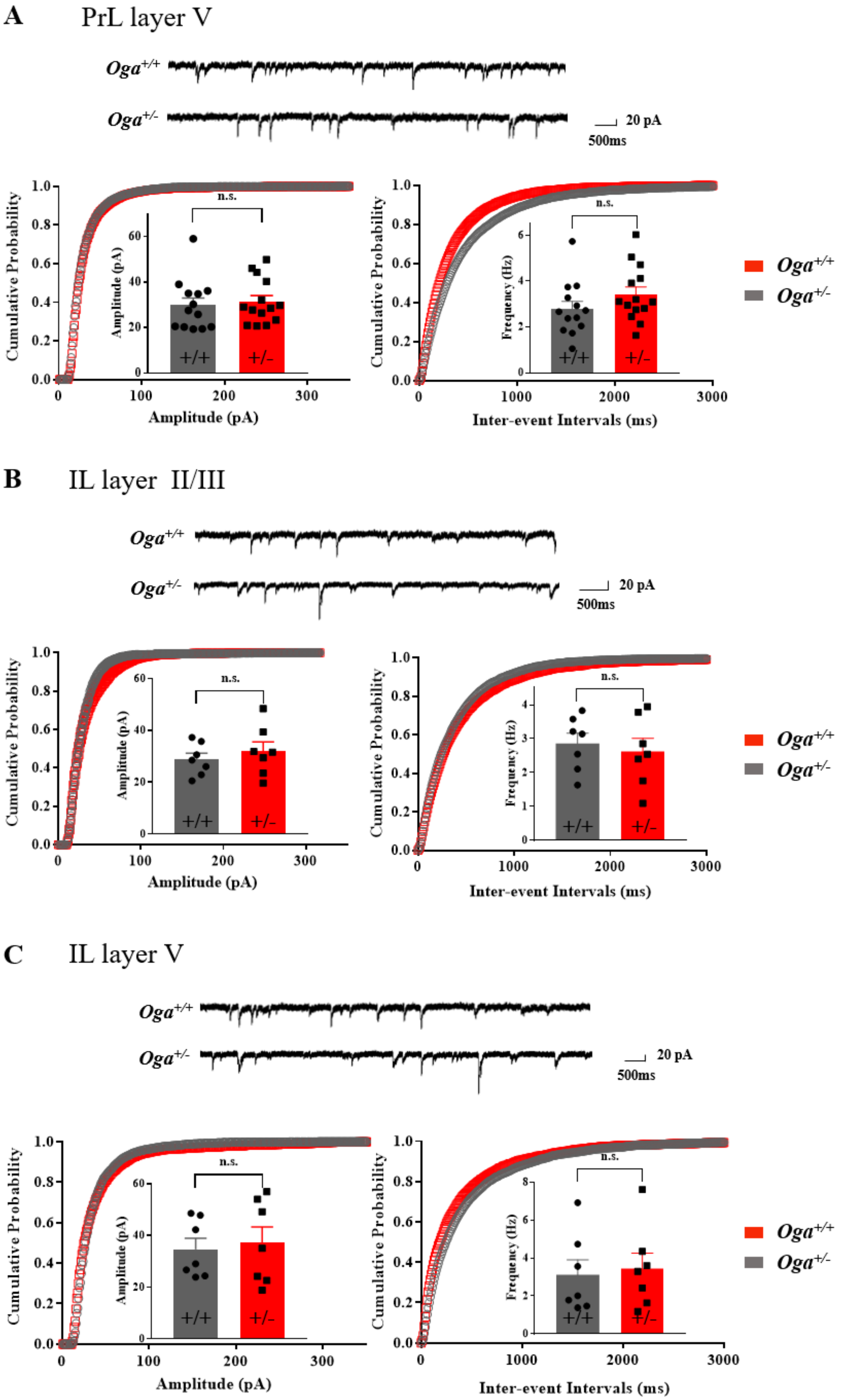
A
PrL layer V
Oga+/+
Oga+/-
20 pA
500ms
Cumulative Probability
Amplitude (pA)
n.s.
+/+
+/-
Inter-event Intervals (ms)
Frequency (Hz)
B
IL layer II/III
C
IL layer V

Supplementary Figure S1. sIPSC recordings from PrL layer V and infralimbic (IL) cortex layer in the mPFC. (A) sIPSC recordings from PrL layer V neurons. Representative traces of sIPSCs (upper), cumulative probability plots of sIPSC amplitude (lower left; $t_{(25)}$=0.3939, P=0.6970) and inter-event intervals (lower right; $t_{(25)}$=1.369, P=0.1832) (*Oga*$^{+/+}$, n=13; *Oga*$^{+/-}$, n=14). **(B)** sIPSC recordings from IL layer II/III neurons. Representative traces of sIPSCs (upper), cumulative probability plots of sIPSC amplitude (lower left; $t_{(12)}$=0.7321, P=0.4782) and inter-event intervals (lower right; $t_{(12)}$=0.4885, P=0.6340) (*Oga*$^{+/+}$, n=7; *Oga*$^{+/-}$, n=7). **(C)** sIPSC recordings from IL layer V neurons. Representative traces of sIPSCs (upper), cumulative probability plots of sIPSC amplitude (lower left; $t_{(12)}$=0.3541, P=0.7294) and inter-event intervals (lower right; $t_{(12)}$=0.3541, P=0.7818) (*Oga*$^{+/+}$, n=7; *Oga*$^{+/-}$, n=7). N.S.: not significant, Student's unpaired *t*-test.

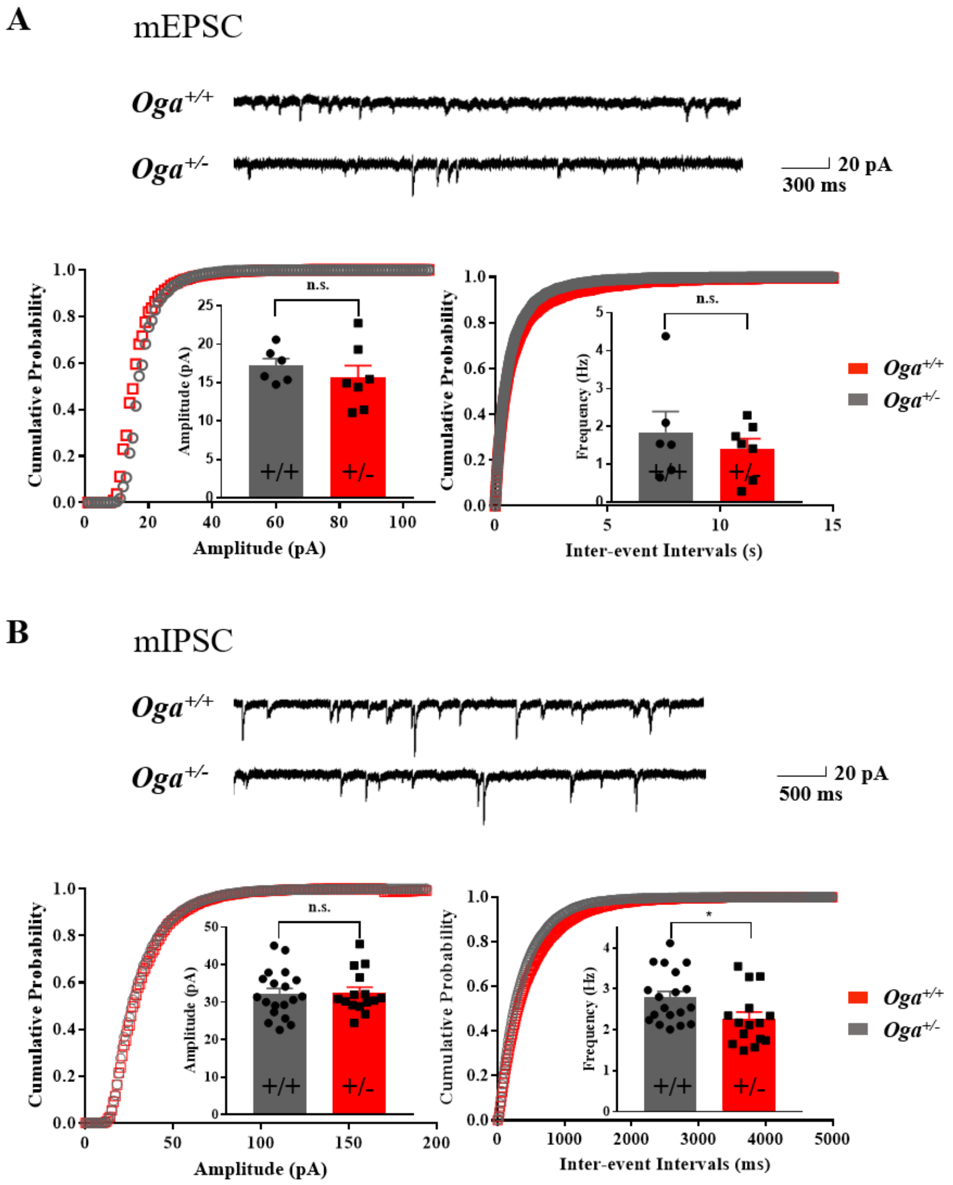

Supplementary Figure S2. Recording mEPSCs and mIPSCs in PrL layer II/III neurons. (A) Representative traces of mEPSCs (upper), cumulative probability plots of mEPSC amplitude (lower left; $t_{(11)}$=0.825, P=0.4269) and inter-event intervals (lower right; $t_{(11)}$=0.7442, P=0.4724) (*Oga*$^{+/+}$, n=7; *Oga*$^{+/-}$, n=7). **(B)** Representative traces of mIPSCs (upper), cumulative probability plots of mIPSC amplitude (lower left; $t_{(32)}$=0.1589, P=0.8747) and inter-event intervals (lower right; $t_{(32)}$=2.359, P=0.0246) (*Oga*$^{+/+}$, n=19; *Oga*$^{+/-}$, n=15). **p*<0.05, n.s.: not significant, Student's unpaired *t*-test.

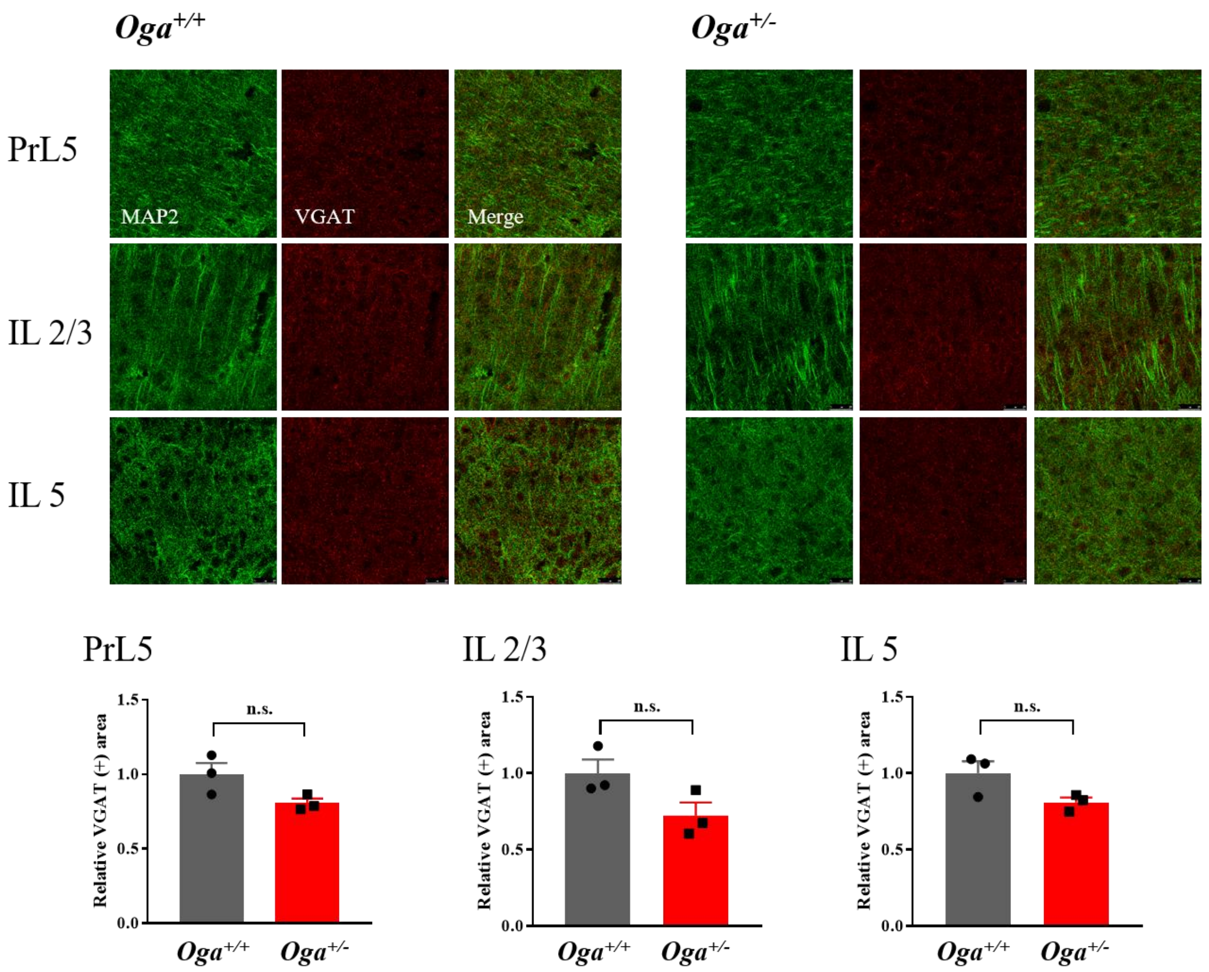

Supplementary Figure S3. Expression levels of VGAT in different layers of mPFC. (Top) Representative immunofluorescence images of VGAT-positive area in PrL layer V, IL layer II/III, and IL layer V. **(Bottom)** Quantification of VGAT-positive area in PrL layer V (*Oga*$^{+/+}$, N=3, n=12; *Oga*$^{+/-}$, N=3, n=11; t(4)=2.364, P=0.0774), IL layer II/III (*Oga*$^{+/+}$, N=3, n=12; *Oga*$^{+/-}$, N=3, n=11; t(4)=2.245, P=0.0881), and IL layer V (Oga+/+, N=3, n=9; Oga+/-, N=3, n=11; t(4)=2.262, P=0.0865). N and n indicate the total number of mice and the number of mPFC slices examined, respectively. n.s.: not significant, Student's unpaired *t*-test.

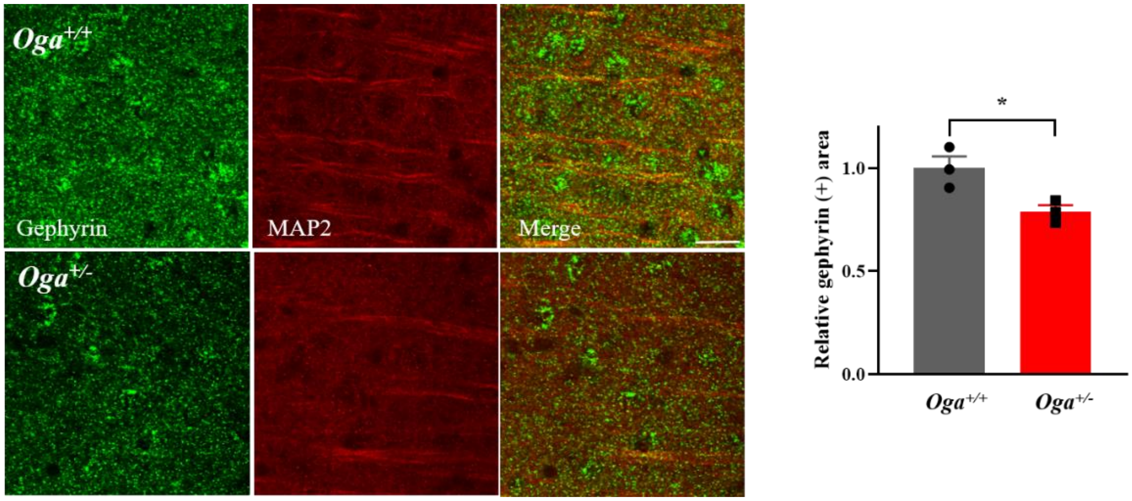

Supplementary Figure S4. Gephyrin expression levels in PrL layer II/III neurons. Representative immunofluorescence images of gephyrin-positive area in the mPFC (left, scale bar, 20 μm), and the graph of normalized gephyrin-positive area (*Oga*$^{+/+}$, N=3, n=11; *Oga*$^{+/-}$, N=3, n=11; t(4)=3.279, P=0.0305). N and n indicate the total number of mice and the number of mPFC slices examined, respectively. *$p<0.05$, Student's unpaired t-test.

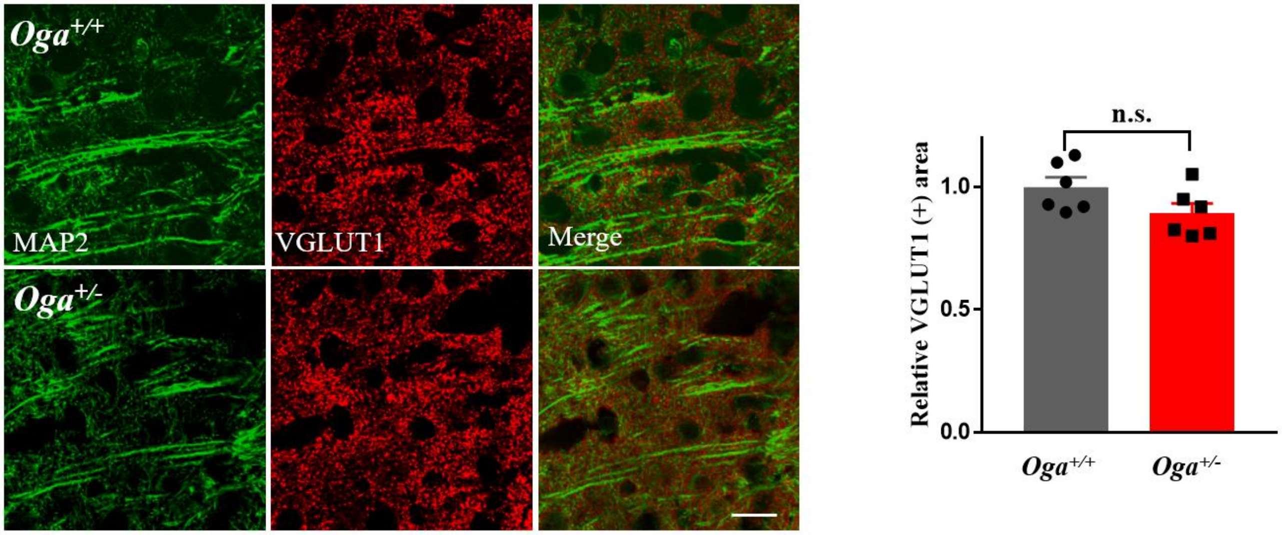

Supplementary Figure S5. VGLUT1 expression levels in PrL layer II/III neurons. Representative immunofluorescence images of VGLUT1-positive area in the mPFC (left, scale bar, 20 μm), and the graph of normalized VGLUT1-positive area (*Oga*$^{+/+}$, N=6, n=24; *Oga*$^{+/-}$, N=6, n=23; $t_{(10)}$=1.861, P=0.0924). N and n indicate the total number of mice and the number of mPFC slices examined, respectively. n.s.: not significant, Student's unpaired *t*-test.

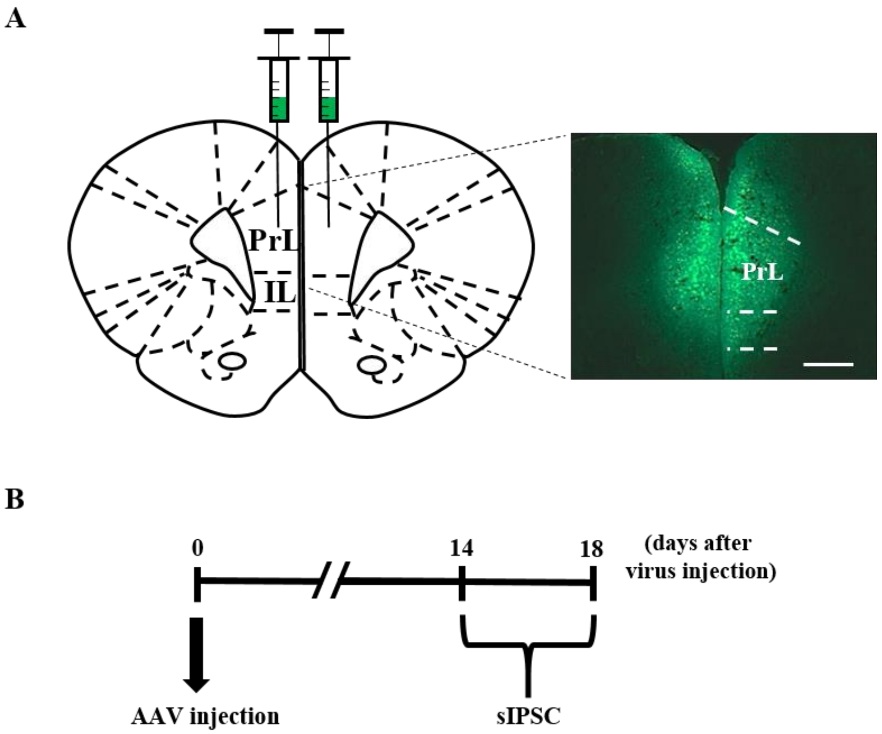

Supplementary Figure S6. Stereotaxic injection of AAV in the PrL layer II/III. (A) Stereotaxic injection site of AAV in the mPFC. The expression of eGFP was confirmed by fluorescence (scale bar, 50 μm). **(B)** Experimental timeline for electrophysiology experiments after virus injection.

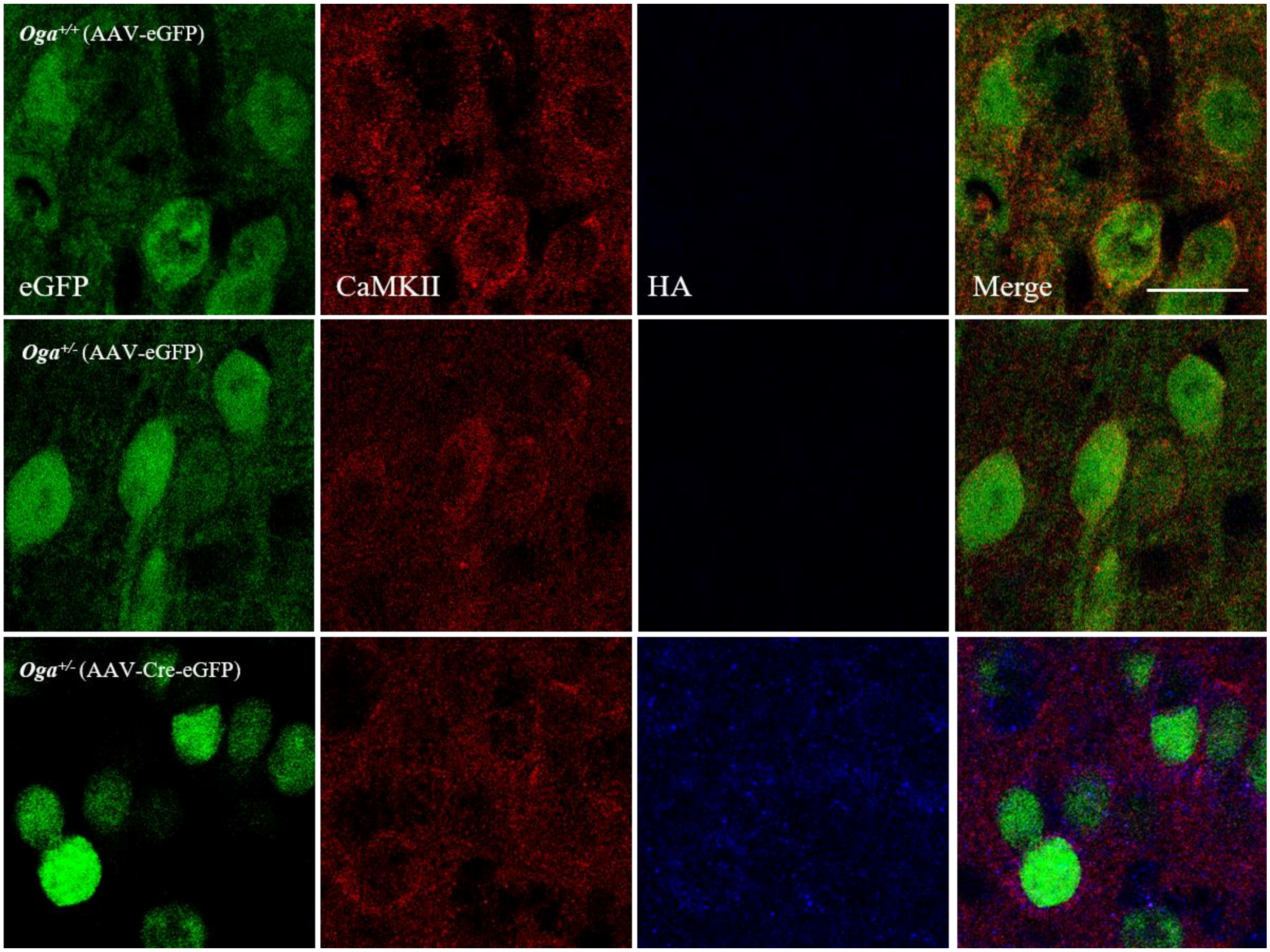

Supplementary Figure S7. Validation of OGA-HA and CaMKII expression in PrL layer II/III. Representative immunofluorescence images of HA-tagged OGA (blue) and CaMKII expression (red) in the PrL layer II/III. Cre-eGFP includes nuclear localization signal, thus its expression is restricted within the nucleus (scale bar, 20 μm).

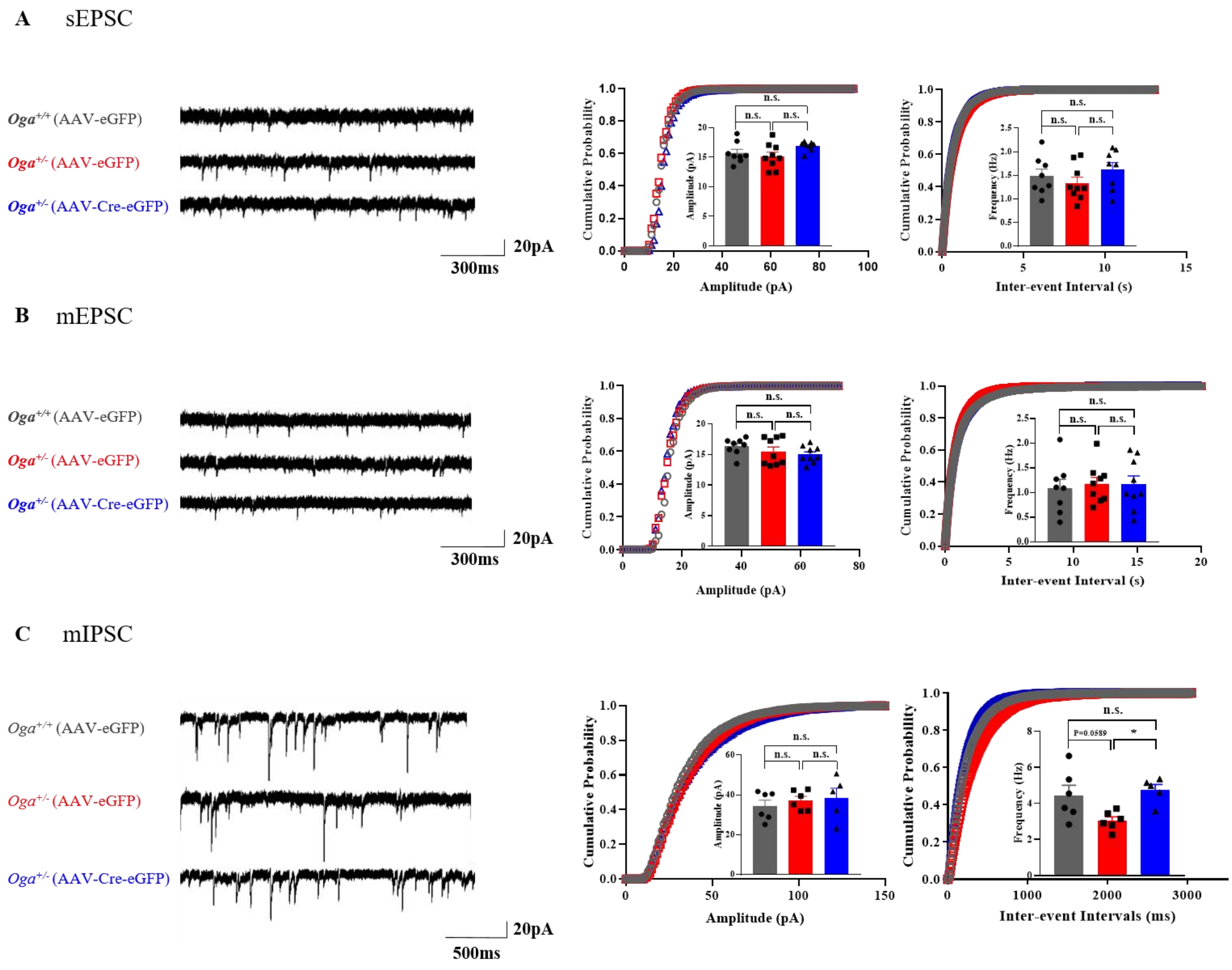

Supplementary Figure S8. Recordings of sEPSC, mEPSC and mIPSC from PrL layer II/III neurons infected with the respective AAV. (A) Representative traces of sEPSCs (left), cumulative probability plots of sEPSC amplitude (middle; $F(2,22)=2.304$, $P=0.1234$) and inter-event intervals (right; $F(2,22)= 1.125$, $P=0.3426$) (AAV-eGFP in *Oga*$^{+/+}$, n=8; AAV-eGFP in *Oga*$^{+/-}$, n=9, AAV-Cre-eGFP in *Oga*$^{+/-}$, n=8). **(B)** mEPSC recordings from IL layer II/III neurons. Representative traces of mEPSCs (left), cumulative probability plots of mEPSC amplitude (middle; $F(2,23)= 1.292$, $P=0.2940$) and inter-event intervals (right; $F(2,23)=0.09155$, $P=0.9128$) (AAV-eGFP in *Oga*$^{+/+}$, n=8; AAV-eGFP in *Oga*$^{+/-}$, n=9, AAV-Cre-eGFP in *Oga*$^{+/-}$, n=9). **(C)** Representative traces of sIPSCs (left), cumulative probability plots of mIPSC amplitude

(middle; $F(2,14)=0.3882$, $P=0.6853$) and inter-event intervals (right; $F(2,14)=0.3882$, $P=0.0215$) (AAV-eGFP in *Oga*$^{+/+}$, n=6; AAV-eGFP in *Oga*$^{+/-}$, n=6, AAV-Cre-eGFP in *Oga*$^{+/-}$, n=5). *$p<0.05$, n.s.: not significant, one-way ANOVA followed by Tukey's multiple comparisons post hoc test.

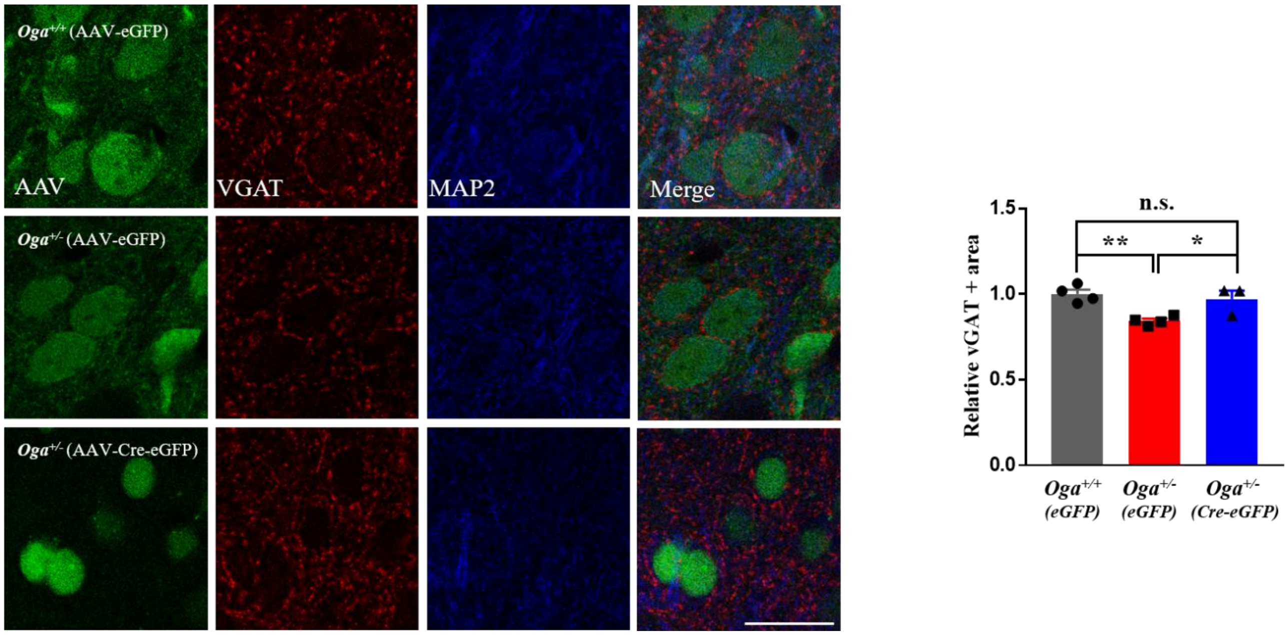

Supplementary Figure S9. OGA overexpression in the mPFC restored the area of VGAT-positive puncta in *Oga*$^{+/-}$ mice. Representative immunofluorescence images of VGAT-positive puncta (red) in the mPFC with the expression of AAV-eGFP or AAV-Cre-eGFP (scale bar, 25 µm), and the graph of normalized VGAT-positive area. (AAV-eGFP in *Oga*$^{+/+}$, N=4, n=16; AAV-eGFP in *Oga*$^{+/-}$, N=4, n=16, AAV-Cre-eGFP in *Oga*$^{+/-}$, N=3 n=10; $F_{(2, 8)}$= 9.116, P=0.0087). N and n indicate the total number of mice and the number of mPFC slices examined, respectively. Cre-eGFP includes nuclear localization signal, thus its expression is restricted within the nucleus. ***p*<0.01, **p*<0.05, n.s.: not significant, one-way ANOVA followed by Tukey *post hoc* test.

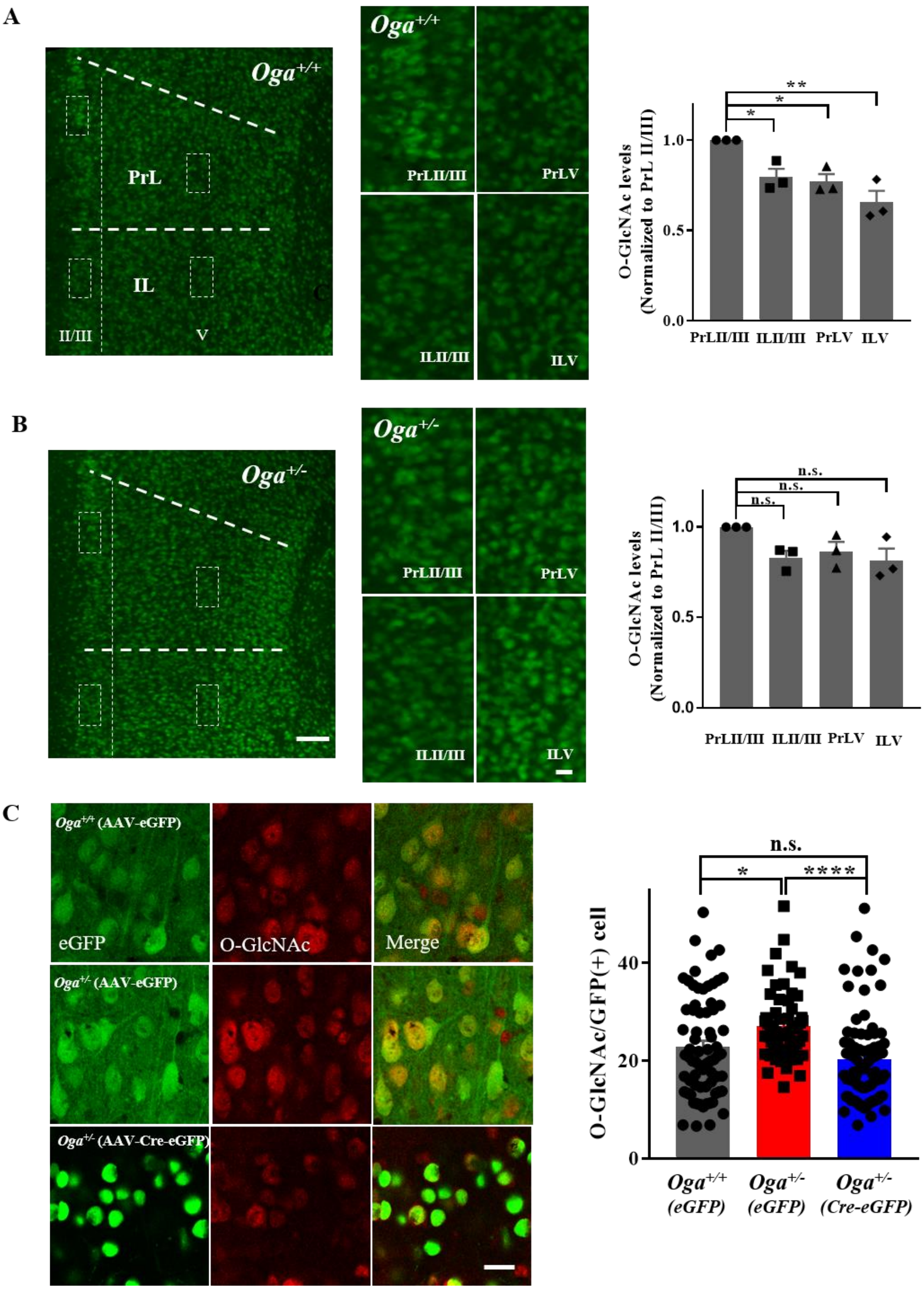
A
Oga+/+
PrL
IL
II/III
V
PrLII/III
PrLV
ILII/III
ILV
O-GlcNAc levels
(Normalized to PrL II/III)
1.0
0.5
0.0
*
*
**
B
Oga+/-
n.s.
C
Oga+/+ (AAV-eGFP)
Oga+/- (AAV-eGFP)
Oga+/- (AAV-Cre-eGFP)
eGFP
O-GlcNAc
Merge
O-GlcNAc/GFP(+) cell
40
20
0
****
Oga+/+ (eGFP)
Oga+/- (eGFP)
Oga+/- (Cre-eGFP)

Supplementary Figure S10. Quantification of O-GlcNAcylation levels in different layers of mPFC. (A) Representative immunofluorescence images of O-GlcNAcylation staining in the mPFC of *Oga*$^{+/+}$ mice (left), enlarged immunofluorescence images of O-GlcNAcylation in PrL layer II/III and V, IL layer II/III and V (middle), and the graph of O-GlcNAcylation levels in different layers of mPFC in *Oga*$^{+/+}$ mice. (F(2, 8)= 10.41, P=0.0039). **(B)** Representative immunofluorescence images of O-GlcNAcylation staining in the mPFC of *Oga*$^{+/-}$ mice (left, scale bar: 100 um), enlarged immunofluorescence images of O-GlcNAcylation in PrL layer II/III and V, IL layer II/III and V (middle, scale bar: 20 um), and the graph of O-GlcNAcylation levels in different layers of mPFC in *Oga*$^{+/-}$ mice (F(3, 8)= 3.332, P=0.0770). **(C)** Immunofluorescence images of O-GlcNAc (red) in the PrL layer II/III with the expression of AAV-eGFP or AAV-Cre-eGFP (left, scale bar: 20um). Cre-eGFP includes nuclear localization signal, thus its expression is restricted within the nucleus. The levels of O-GlcNAcylation were quantified from GFP-positive neurons (right, AAV-eGFP in *Oga*$^{+/+}$, n=70; AAV-eGFP in *Oga*$^{+/-}$, n=60, 82 AAV-Cre-eGFP in *Oga*$^{+/-}$, n=80; F(2, 207)=9.628, P=0.0001). ****p<0.0001, **p<0.01, *p<0.05, n.s.: not significant, one-way ANOVA followed by Tukey *post hoc* test.

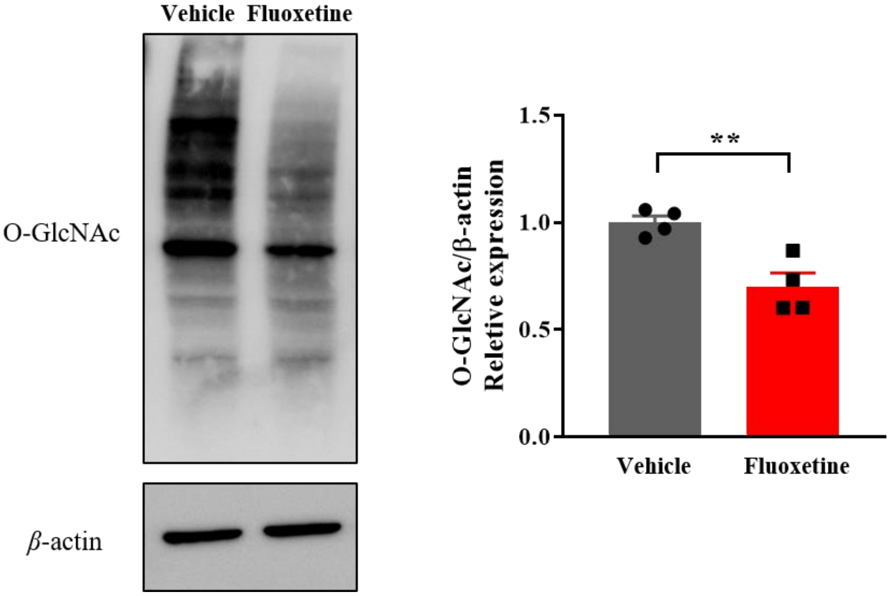

Supplementary Figure S11. The Effect of acute fluoxetine treatment on O-GlcNAcylation levels in the PFC. A change in O-GlcNAcylation levels was examined 30 min after fluoxetine treatment (20 mg/kg, i.p.). The graph of O-GlcNAcylation levels normalized to vehicle (vehicle n=4, fluoxetine n=4; $t_{(6)}$=4.243, P=0.0054). ***p*<0.01, n.s.: not significant, unpaired Student's *t*-test.

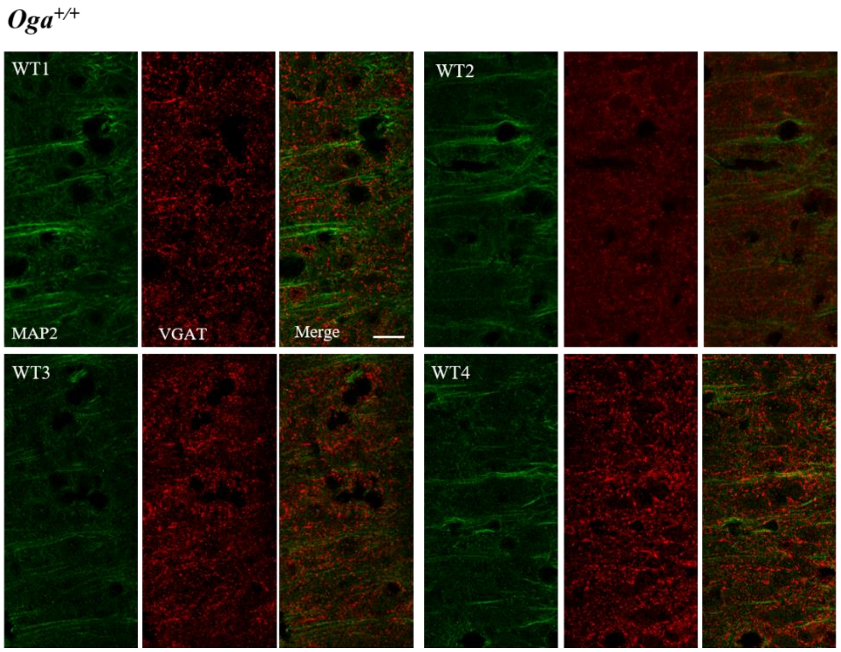

Oga+/+
WT1
MAP2
VGAT
Merge
WT2
WT3
WT4

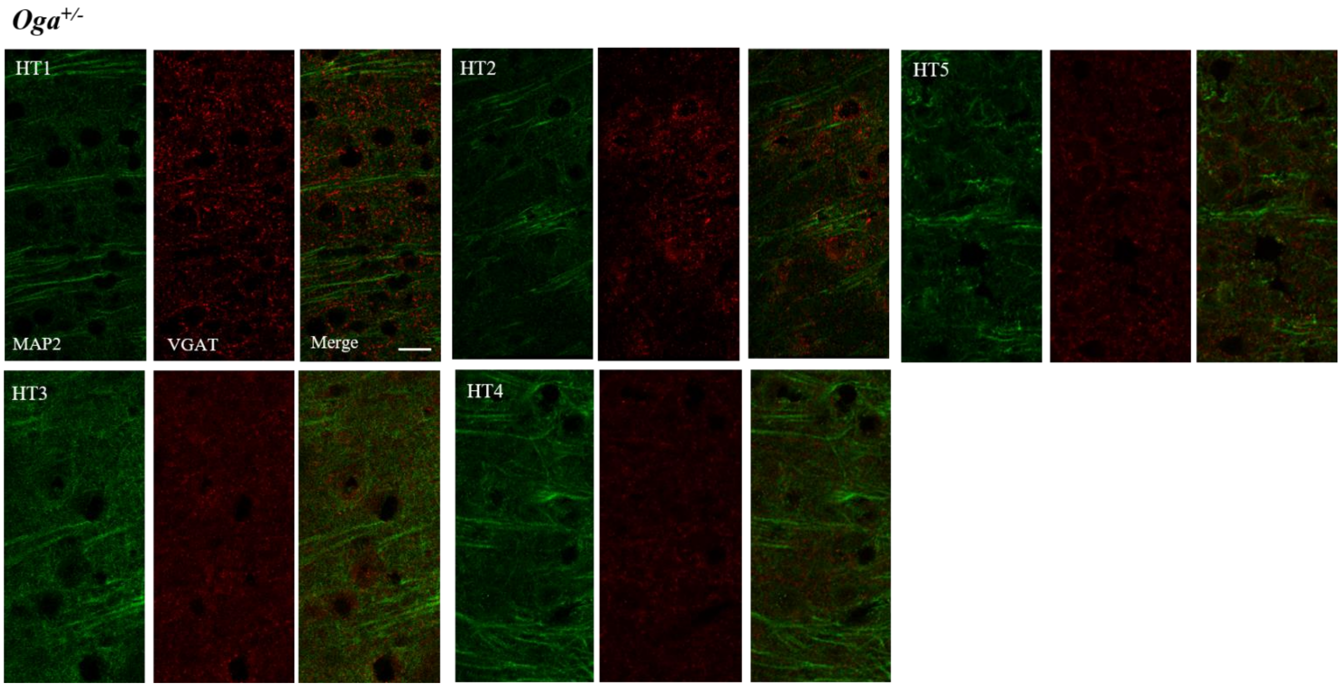

Oga+/-
HT1
MAP2
VGAT
Merge
HT2
HT5
HT3
HT4

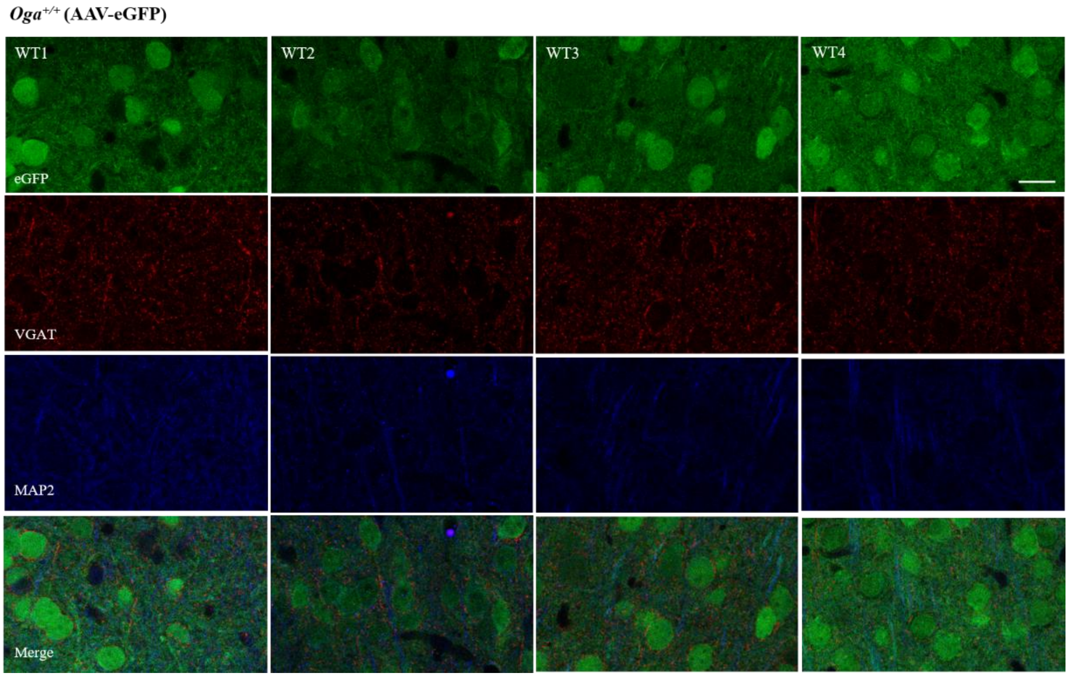
Oga+/+ (AAV-eGFP)
WT1
WT2
WT3
WT4
eGFP
VGAT
MAP2
Merge

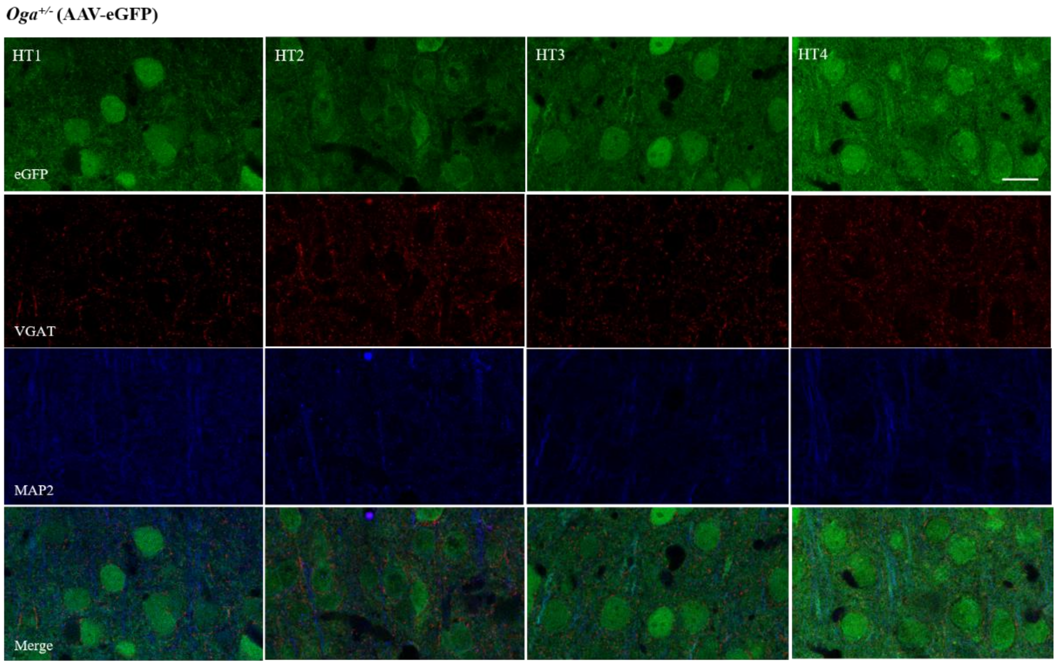
Oga+/- (AAV-eGFP)
HT1
HT2
HT3
HT4
eGFP
VGAT
MAP2
Merge

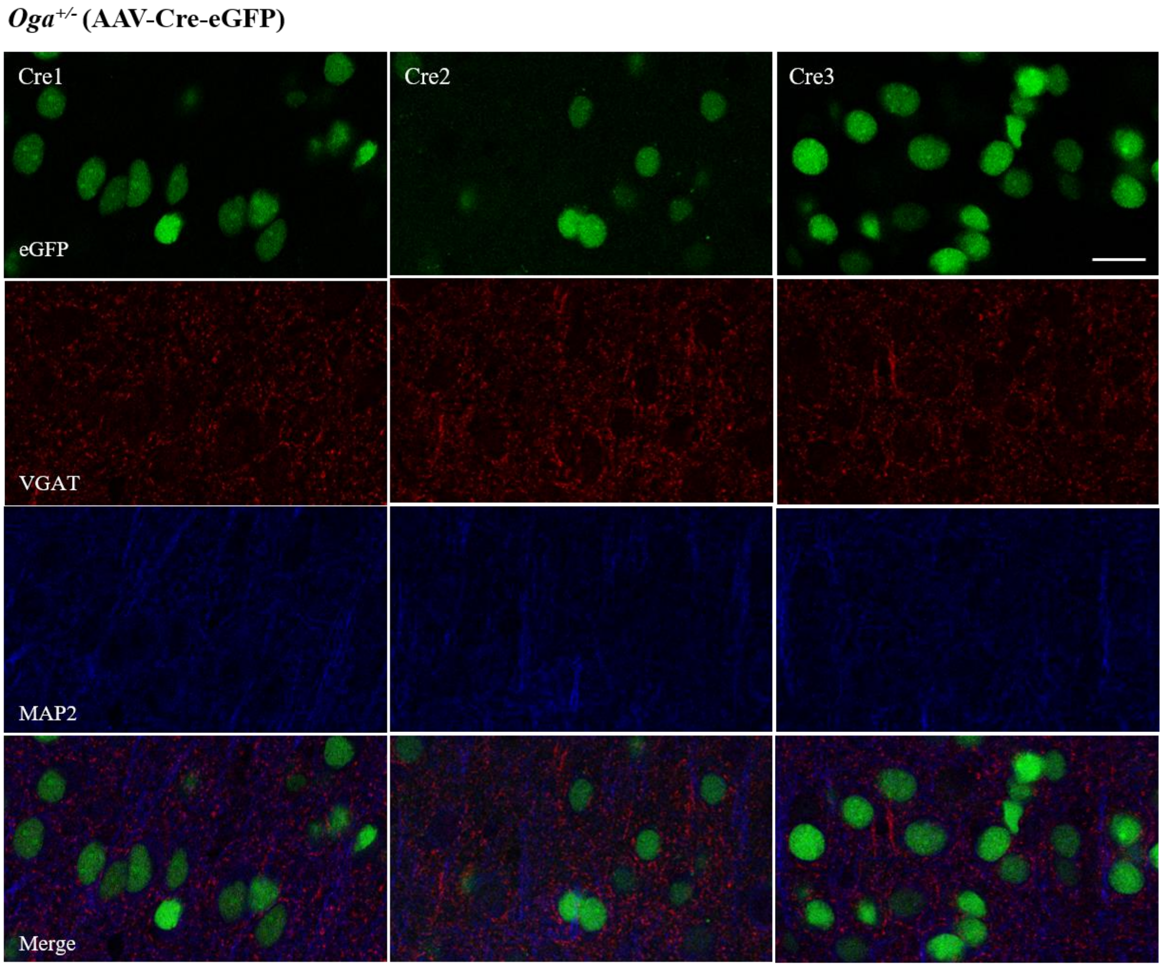

Supplementary Figure S12. Collection of raw images of VGAT staining used for the quantification of VGAT expression levels.

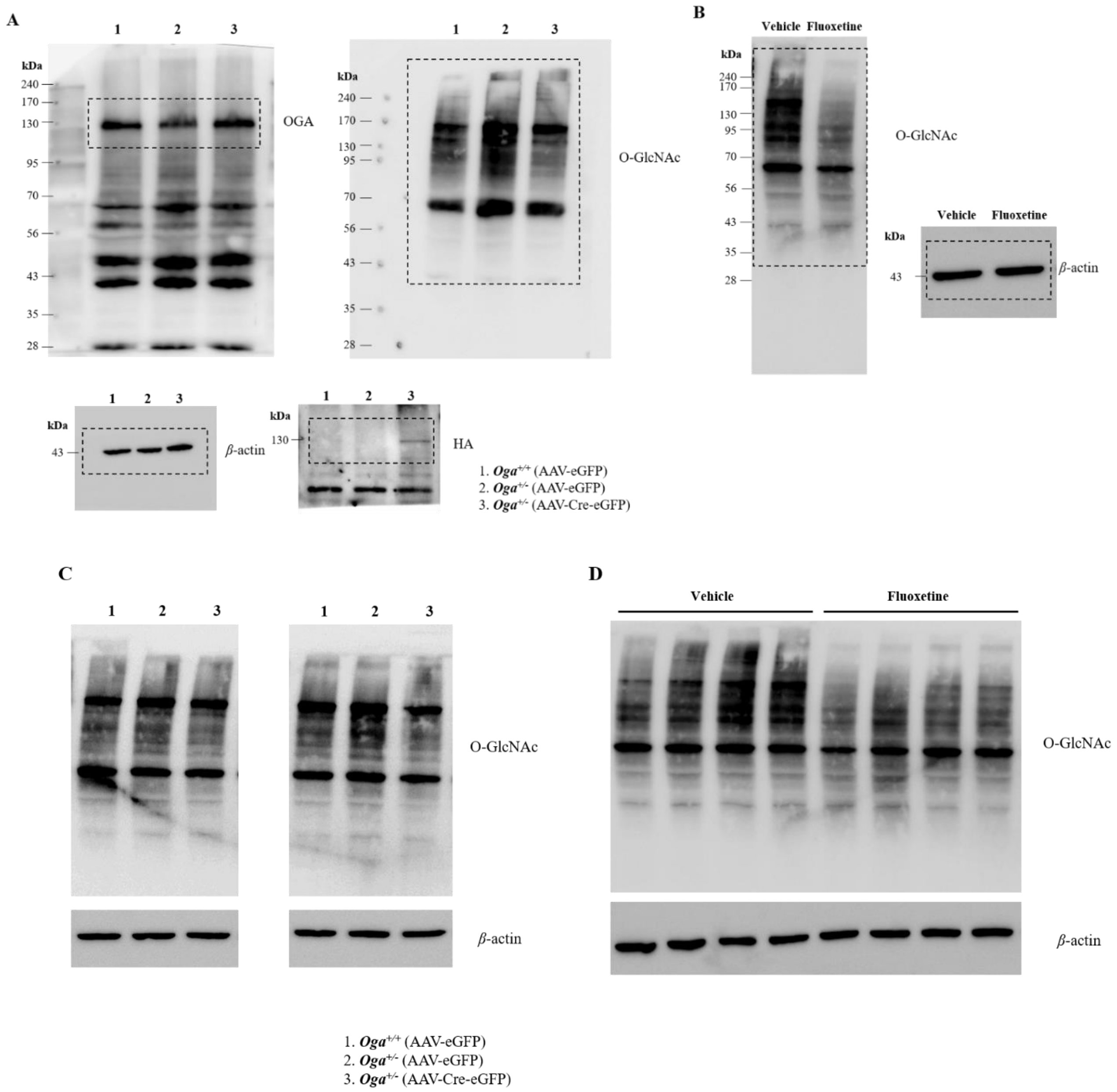

Supplementary Figure S13. Full-length immunoblots. (A, B) The original images for the immunoblots in (A) Figure 3B and (B) Supplementary Figure S11 are shown. Dashed boxes indicate the cropped areas presented in the main and supplementary figures. **(C, D)** The original images of additional immunoblots used for the quantification of O-GlcNAcylation levels.
